# A Comparative Transcriptome Analysis Reveals the Molecular Mechanisms That Underlie Somatic Embryogenesis in *Peaonia ostii* ‘Fengdan’

**DOI:** 10.3390/ijms231810595

**Published:** 2022-09-13

**Authors:** Huiting Ci, Changyue Li, Theint Thinzar Aung, Shunli Wang, Chen Yun, Fang Wang, Xiuxia Ren, Xiuxin Zhang

**Affiliations:** 1 Key Laboratory of Biology and Genetic Improvement of Horticultural Crops, Institute of Vegetables and Flowers, Chinese Academy of Agricultural Sciences, Ministry of Agriculture and Rural Affairs, Beijing 100081, China; 2 National Agricultural Science and Technology Center, Chengdu 610213, China

**Keywords:** somatic embryogenesis, tree peony, transcriptome analysis, hormone network, stress response, epigenetic modifications

## Abstract

Low propagation rate is the primary problem that limits industry development of tree peony. In this study, a highly efficient regeneration system for tree peony using somatic embryogenesis (SE) was established. The transcriptomes of zygotic embryo explants (S0), non-embryonic callus (S1), embryonic callus (S2), somatic embryos (S3), and regenerated shoots (S4) were analyzed to determine the regulatory mechanisms that underlie SE in tree peony. The differentially expressed genes (DEGs) were identified in the pairwise comparisons of S1-vs-S2 and S1-vs-S3, respectively. The enriched DEGs were primarily involved in hormone signal transduction, stress response and the nucleus (epigenetic modifications). The results indicated that cell division, particularly asymmetric cell division, was enhanced in S3. Moreover, the genes implicated in cell fate determination played central roles in S3. Hormone signal pathways work in concert with epigenetic modifications and stress responses to regulate SE. *SERK*, *WOX9*, *BBM*, *FUS3*, *CUC*, and *WUS* were characterized as the molecular markers for tree peony SE. To our knowledge, this is the first study of the SE of tree peony using transcriptome sequencing. These results will improve our understanding of the molecular mechanisms that underly SE in tree peony and will benefit the propagation and genetic engineering of this plant.

## 1. Introduction

Tree peony (*Paeonia* Sect. Moutan DC.) is an important ornamental plant that is native to China. The plant is also known for its edible and medicinal values. Cortex Moutan is a famous Chinese traditional medicine listed in the Chinese Pharmacopeia [1] that is widely used to treat arthritis, traumatic injury, tumor, and nerve defects [2,3,4,5]. Tree peony is also recognized as an important oil plant owing to its rich content of unsaturated fatty acids, particularly α-linolenic acid [6,7]. Currently, the creation of new varieties and the mass production of uniform seedlings is an extremely urgent process owing to the rapid development of tree peony industry. However, it takes more than 10 years to breed a new variety and propagation coefficient of the traditional methods (division and grafting) is relatively low [8], which hinders the breeding process and industrial development.

Plant regeneration via somatic embryogenesis (SE) has substantial potential for mass multiplication, and it has been widely used for commercial plant micropropagation and transgenic plant production [9]. However, the regenerative capacity varies among different genotypes [10,11]. Thus, there is an urgent need to improve this situation. A series of studies were conducted on the plant regeneration of tree peony that has primarily focused on the following explants, including zygotic embryos, cotyledons, and hypocotyls [10,12,13]. Shoot organogenesis has also been successfully induced from the young leaves of peony [14]. Even though there has been some process in the plant regeneration of tree peony, the process still depends on the available genotypes. Moreover, abnormalities and low efficiency are still serious problems in the regeneration of tree peony plants [10]. Elucidation of the underling molecular basis will significantly help to solve these problems. However, to date, little research has been reported on the molecular mechanism of plant regeneration in tree peony.

The regenerative capacity of plants is always controlled by the endogenous factors that include developmental stages and reactive oxygen species (ROS) levels, as well as the culture medium and environmental conditions [11,15]. Auxin gradients established by polar transport and biosynthesis in the localized region are vital for the induction of SE [16]. *SOMATIC EMBRYOGENESIS RECEPTOR-LIKE KINASE* (*SERK*), *BABY BOOM* (*BBM*), *LEAFY COTYLEDON* (*LEC*), *FUSCA3* (*FUS3*), *CUP SHAPED COTYLEDONS* (*CUC*), and *WUSCHEL* (*WUS*) are involved in the induction of SE [17]. The specification of stem cell fate and the construction of shoot apical meristems (SAMs) are vital for plant regeneration [18]. *WUS* specifies the fate of stem cells and functions as the master of SAM [19]. Cytokinin is the regulator of SAM, and this response is mediated by *B-type Arabidopsis response regulators* (*B-type ARRs*) [11,20]. It has been reported that four *B-type ARRs* (*ARR1*, *ARR2*, *ARR10*, and *ARR12*) play essential roles in the construction of SAM and shoot regeneration [20,21]. Moreover, epigenetic reprogramming substantially regulates the transition of cell fate, formation of callus, induction of SE, and establishment of SAM [22,23,24,25,26]. An inhibitor of histone deacetylases can induce SE through the de-repression of genes related to SE [27,28]. Histone H3 lysine 27 trimethylation (H3K27me3) modification engages in cross-talk with hormones to regulate the establishment of embryogenic competence in *Arabidopsis thaliana* [15]. In addition, ROS homeostasis is involved in the de novo initiation of shoots and its closely related to the ability of plants to regenerate [29]. Peroxidases are always enhanced in SE and are used as the markers of SE capacities [30,31].

Even though much process has been made in understanding the underlying mechanisms of SE, our understanding of the molecular networks of SE is still limited, particularly in tree peony. An efficient protocol for shoot regeneration via SE was established in tree peony in this study using the zygotic embryo as an explant. Based on this protocol, samples in five key stages, including the zygotic embryo explant (S0), callus formation (S1), embryonic callus (S2), somatic embryos (S3), and the regenerated shoots (S4) were collected for analysis by transcription sequencing. The transcriptome dynamics reveal a regulatory network that underlie hormones, chromatin modification, and ROS homeostasis to jointly induce SE. Our results provide a valuable reference for the molecular mechanisms that underly SE.

## 2. Results

### 2.1. Morphological and Histological Analysis of the Entire Plant Regeneration Process

**Figure 1:**
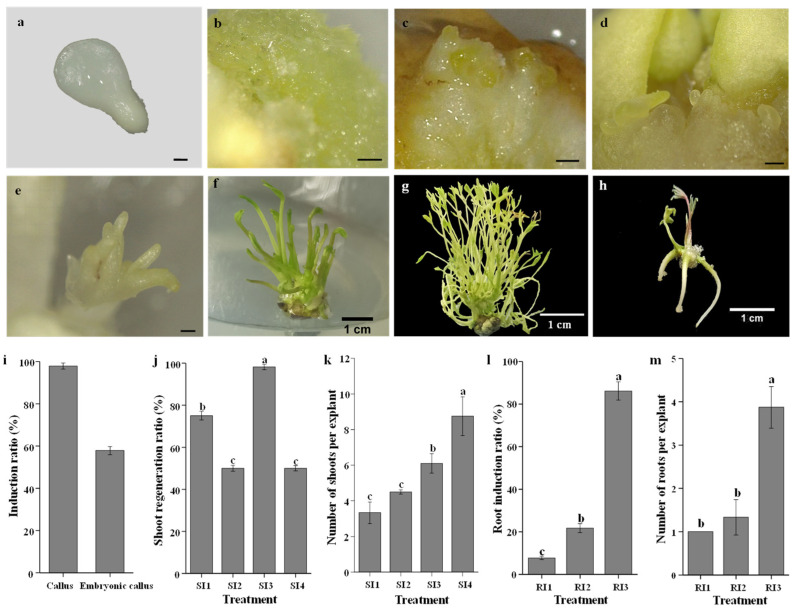
Whole process of plant regeneration via somatic embryogenesis and the related indexes. (**a**) Zygotic embryo explant (S0), (**b**) Non-embryonic callus (S1), (**c**) Embryonic callus (S2), (**d**) Somatic embryos (S3), (**e**–**g**) The regenerated shoots (S4), (**h**) The regenerated roots, (**i**) Induction ratio of callus and embryonic callus, (**j**) Shoot regeneration ratio in different treatments, (**k**) Number of shoots per explant in different treatments, (**l**) Root induction ratio in different treatments, (**m**) Number of roots per explant in different treatments. Scale bar of (**a**–**e**) is 500 μm and scale bar of (**f**–**h**) is 1 cm. Different lowercase letters in (**i**–**m**) indicate significant differences among treatments (Duncan’s test at *p* < 0.05 after analysis of variance). The medium in (**a**–**d**) is WPM + 4.0 mg·L^−1^ phytagel + 30 g·L^−1^ sucrose + 3.0 mg·L^−1^ 6-BA and 1.0 mg·L^−1^ NAA; the medium for shoot development in (**e**–**g**) is SI1–4, SI1 (WPM + 4.0 mg·L^−1^ phytagel + 30 g·L^−1^ sucrose + 0.5 mg·L^−1^ 6-BA + 0.5 mg·L^−1^ GA_3_); SI2 (WPM + 4.0 mg·L^−1^ phytagel + 30 g·L^−1^ sucrose + 0.5 mg·L^−1^ 6-BA + 1.0 mg·L^−1^ GA_3_); SI3 (WPM + 4.0 mg·L^−1^ phytagel + 30 g·L^−1^ sucrose + 1.0 mg·L^−1^ 6-BA + 0.5 mg·L^−1^ GA_3_); SI4 (WPM + 4.0 mg·L^−1^ phytagel + 30 g·L^−1^ sucrose + 1.0 mg·L^−1^ 6-BA + 1.0 mg·L^−1^ GA_3_); and the medium for root induction in (**h**) is RI1-3. RI1 (WPM + 4.0 mg·L^−1^ phytagel + 30 g·L^−1^ sucrose + 3.0 mg·L^−1^ 6-BA +3.0 mg·L^−1^ IBA + 2.0 mg·L^−1^ caffeic acid), RI2 (WPM + 4.0 mg·L^−1^ phytagel + 30 g·L^−1^ sucrose + 3.0 mg·L^−1^ 6-BA +3.0 mg·L^−1^ IBA + 1.0 mg·L^−1^ acetylsalicylic acid), RI3 (WPM + 4.0 mg·L^−1^ phytagel + 30 g·L^−1^ sucrose + 3.0 mg·L^−1^ 6-BA + 2.0 mg·L^−1^ IBA + 1.0 mg·L^−1^ acetylsalicylic acid).

An efficient system for callus induction, SE, shoot development, and root induction was established in this study (Figure 1a–h). The results showed that MS media supplemented with 3.0 mg·L^−1^ 6-BA and 1.0 mg·L^−1^ NAA could successfully induce embryonic callus and somatic embryos in tree peony (*Peaonia ostii* ‘Fengdan’), WPM medium supplemented with 1.0 mg·L^−1^ 6-BA and 0.5 mg·L^−1^ GA_3_ (SI3) enhanced shoot development from somatic embryos, and WPM medium supplemented with 3.0 mg·L^−1^ 6-BA, 2.0 mg·L^−1^ IBA, and 1.0 mg·L^−1^ acetylsalicylic acid in concert with a 15-day period of cold treatment, accelerated the induction of roots. The callus was initiated after 2–4 weeks of culture (Figure 1b). The induction ratio of non-embryonic callus was 97.92% (Figure 1i). Smooth and light-yellow embryonic calli were obtained after 2 months of culture (Figure 1c). The induction ratio of embryonic callus was 57.88%. The somatic embryos, including different stages from the globular to cotyledonary stages, were formed after 3 months of culture with a newly constructed SAM (Figure 1d). Subsequently, the somatic embryos were transferred to four types of shoot induction medium for shoot development. Shoots were derived from those embryos and grew large after an additional 1–2 months of culture (Figure 1e–g). The regenerated shoots developed well in the SI3 medium, which resulted in the highest shoot regeneration ratio (98.2%), followed by SI1 with an induction ratio of 75% (Figure 1j). SI4 had the largest number of regenerated shoots per explant (8.75), followed by SI3 medium (6.10) and then SI2 medium (4.50) (Figure 1k). The regenerated shoots were cultured in RI1-3 medium for root induction. The roots had regenerated after 2 months of culture (Figure 1h). The root induction ratio was significantly enhanced in R3 medium with an induction ratio of 86.11% (Figure 1l). The number of roots per explant was the highest in R3 medium (3.87) (Figure 1m).

**Figure 2:**
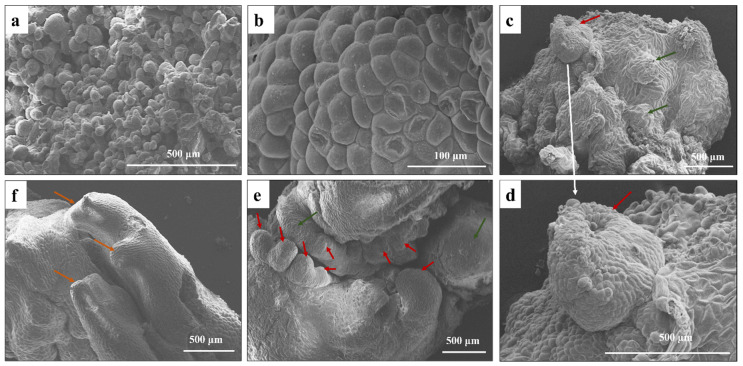
Scanning electron microscope (SEM) observation of shoot regeneration process. (**a**) Non-embryonic callus (S1), (**b**) Embryonic callus (S2), (**c**–**e**). Somatic embryos (S3) with established SAMs, (**f**) The regenerated shoots (S4). Green arrows indicated the established SAMs, red arrows marked somatic embryos, and yellow arrow means regenerated shoots.

We observed the morphology of the whole SE process using a stereomicroscope and found that there were four key stages involved in the whole process (Figure 2), including S1, the formation of non-embryonic callus; S2, the transition of cell fate to embryonic cell and the induction of embryonic callus; S3, the formation of somatic embryos and the de novo construction of SAM; and S4, the regenerated shoots from the somatic embryos. Observation by stereomicroscope and SEM showed that the somatic cells acquired pluripotency and formed calli after 2–4 weeks of culture (Figure 2a). These non-embryonic calli were soft, uneven, and white. The cells were disorganized. After 1–2 months of culture, certain sites of the normal callus gradually became smooth, and the cells became better organized (Figure 2b). By this time, the cell fate had changed from normal callus cells to embryonic cells. After 3 months of culture, somatic embryos in different stages were observed, and SAM was established (Figure 2c–e). Cells in the somatic embryos were all highly organized. Their surfaces were very smooth and glossy. After another 1–2 months of culture, the regenerated shoots had developed well in the SI medium (Figure 2f).

### 2.2. Illumina Sequencing, De Novo Transcriptome Assembly, Functional Annotation, and Classification

Samples including the zygotic embryo explants at S0, the calli or embryos at S1–3, and the regenerated shoots at S4 were collected for transcriptomic analyses. In absence of reference genome, reads were filtered and used for the de novo transcriptome assembly. 32.1 Gb nucleotides were obtained by Illumina sequencing. The resulting transcriptome was 219.1 Mb size. After the removal of ambiguous reads, adapter sequences, and the low-quality reads, a total of 131,496 unigenes were assembled in tree peony with average sequence length of 948 bp, N50 length of 1666 bp, and GC percentage of 40.04% (Table 1).

BUSCO (Benchmarking Universal Single-Copy Orthologs) assessment results showed that the transcriptome was well assembled and the integrity was good (Figure S1a). The distribution of the unigene length is shown in Figure S1b. A total of 131,496 unigenes were identified (Table S1). The number of unigenes decreased as the size of sequences increased. The unigenes with more than 3000 bp nucleosides comprised 4.56%, unigenes greater than 1000 bp comprised 32.39%, and unigenes greater than 500 bp comprised 51.52%. In addition, 46,659 coding sequences (CDS) were predicted. The greatest numbers of CDS were 300–400 bp. The rates of CDS that were larger than 3000 bp, 1000 bp, and 500 bp were 3.41%, 42.99%, and 83.89%, respectively. A comparison of the unigenes was annotated with the homologous sequences of other plant species, and the best match among these nucleotide sequences was *Vitis vinifera* (19.05%) (Figure S1c).

About 43.91% of all assembled unigenes were annotated by the NCBI non-redundant protein sequences (NR) database, 37.10% were annotated by NCBI non-redundant nucleotide sequences (NT) database; 31.98% were annotated by Swiss-Prot; 34.29% were annotated by Kyoto Encyclopedia of Genes and Genomes(KEGG); 34.10% were annotated using Eukaryotic Orthologous Groups of proteins/Clusters of Orthologous Groups of proteins (KOG); 23.08% were annotated in the prediction of protein structure domain database (Pfam); and 33.23% were annotated by Gene Ontology (GO) (Table 2). A total of 27,822 unigenes (21.15%) were annotated by all five public databases including NR, Swiss-Prot, KEGG, KOG, and Pfam, and more than 40% of all assembled unigenes were annotated by at least one database (Figure S2). Unigenes from NR database were also annotated by KEGG, Swiss-Prot, Pfam, or KOG with the proportion of 34.07%, 31.80%, 30.04%, 33.92%, respectively. A total of 37,345 (28.40%) were annotated by both KEGG and Swiss-Prot, 33,667 (25.60%) were annotated by both KEGG and Pfam, 39,876 (30.32%) were annotated by both KEGG and KOG, 32,266 (24.54%) were annotated by both Swiss-Prot and Pfam, 37,820 (28.76%) were annotated by both Swiss-Prot and KOG, and 33,858 (25.75%) were annotated by both Pfam and KOG (Figure S2). In addition, 31.89% genes from NR database were also annotated by NT (Figure S2). A total of 33,793 (25.70%) unigenes were annotated by both NT and Pfam, and 33,891 (25.77%) unigenes were annotated by both NT and Swiss-Prot (Figure S2).

**Figure 3:**
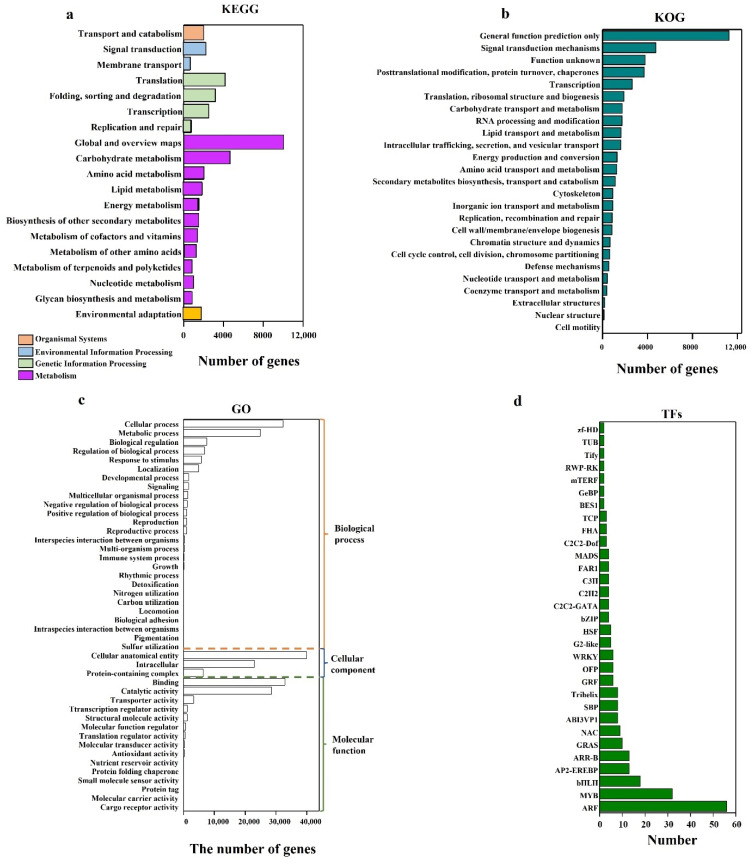
Functional analysis of the genes identified. (**a**) KEGG pathways distribution. (**b**) Gene Ontology (GO) assignments. (**c**) KOG functional classification. (**d**) Transcription factor (TF) categories of tree peony DEGs.

KEGG is an important database used for understanding the gene functions of various biological systems. A total of 43,587 unigenes were assigned to KEGG pathways, divided into 5 main categories with 19 subcategories: ‘metabolism’ (61.04%) was dominant, followed by ‘genetic information processing’ (24.11%), ‘environmental information processing’ (6.44%), ‘cellular processes’ (4.57%), and ‘organismal systems’ (3.84%) (Figure 3a). ‘Metabolism’ had 11 sub-categories, among which, ‘global and overview maps’ with 10,033 unigenes (37.71%) accounted for the largest, followed by ‘carbohydrate metabolism’ (17.44%). ‘Genetic information processing’ included 4152 unigenes (39.51%) assigned to ‘translation’, 3118 (29.67%) assigned to ‘folding, sorting and degradation’, 2471 (23.52%) assigned to ‘transcription’, and 767 (7.30%) assigned to ‘replication and repair’. The results indicated that ‘metabolism’ and ‘genetic information processing’ were closely related to SE.

The KOG functional annotation of tree peony unigenes showed that a total of 44,846 unigenes were classified into 25 KOG functional groups, among which the top 8 categories were ‘general function prediction only’ (25.10%), ‘signal transduction mechanisms’ (10.58%), ‘function unknown’ (8.43%), ‘posttranslational modification, protein turnover’ (8.21%), ‘transcription’ (5.84%), ‘translation, ribosomal structure and biogenesis’ (4.21%), ‘carbohydrate transport and metabolism’ (3.91%), and ‘RNA processing and modification’ (3.88%) (Figure 3b). Additionally, 910 unigenes in ‘cytoskeleton’ accounted for 2.03%, 661 unigenes in ‘chromatin structure and dynamics’ occupied 1.42%, and 635 unigenes in ‘cell cycle control, cell division, chromosome partitioning’ took up 1.42%.

Gene ontology (GO) is used as a convenient tool for gene annotation as well as gene identification. Here, 231,222 unigenes annotated by GO were divided into three main categories: ‘biological process’ (40.03%), ‘cellular component’ (30.00%), and ‘molecular function’ (29.97%). Among the biological processes, ‘cellular process’ (34.82%) and ‘metabolic process’ (26.94%) were dominant, followed by ‘biological regulation’ (8.22%), ‘regulation of biological process’ (7.40%), ‘response to stimulus’ (6.26%), and ‘localization’ (5.34%) (Figure 3c). ‘Cellular process’ included ‘cellular anatomical entity’ (57.55%), ‘intracellular’ (33.17%), and ‘protein-containing complex’ (9.27%). ‘Binding’ (47.46%) and ‘catalytic activity’ (41.29%) were dominant in ‘molecular function’. These results obtained from KEGG, GO, and KOG analysis could be helpful for clarifying the mechanisms underlying SE in tree peony.

Among 1939 transcription factors (TFs), 250 were substantially up-regulated in embryonic callus (S2) and somatic embryos (S3) compared with those of non-embryonic callus (S1), including 56 ARFs, 32 MYB, 18 bHLH, 13 ARR-B, 13 AP2-EREBP, 10 GRAS, 9 NAC, 8 ABI3VP1, 8 SBP, 8 Trihelix, 6 GRF, 6 OFP, 6 WRKY, 5 G2-like, 4 HSF, 4 bZIP, 4 C2C2-GATA, 4 C2H2, 4 C3H, 4 FAR1, 4 MADS, and 23 other TFs (Figure 3d), indicating that these TFs may be closely related with SE.

### 2.3. Analysis of DEGs

**Figure 4:**
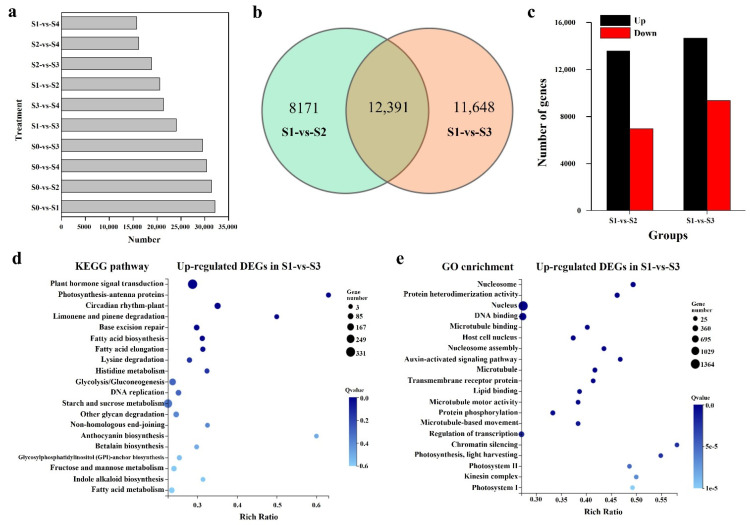
The number of DEGs in 10 groups and KEGG and GO enrichment analysis of the DEGs in S1-vs.-S3. (**a**) Number of DEGs in 10 groups. (**b**) Venn diagram showing overlap between S1-vs.-S2 and S1-vs.-S3. (**c**) The number of up-regulated and down-regulated DEGs in S1-vs.-S2 and S1-vs.-S3. (**d**) KEGG enrichment of the up-regulated DEGs in S1-vs.-S3. (**e**) GO enrichment of the up-regulated DEGs in S1-vs.-S3.

A total of 31,455, 29,562, 30,372, 32,176, 20,561, 24,039, 15,734, 18,826, 16,128, and 21,358 DEGs were found in S0-vs.-S2, S0-vs.-S3, S0-vs.-S4, S1-vs.-S0, S1-vs.-S2, S1-vs.-S3, S1-vs.-S4, S2-vs.-S3, S2-vs.-S4, and S3-vs.-S4, respectively (Figure 4a and Table S2). S0-vs.-S2, and S0-vs.-S3 shared 20,978 unigenes, while S0-vs.-S1, S0-vs.-S2, and S0-vs.-S3 shared 16,802 unigenes (Figure S3). S1-vs.-S2 and S1-vs.-S3 shared 12,391 unigenes (Figure 4b). The identification of the regulatory pathways of SE was based on the analysis of DEGs in S1-vs.-S2 and S1-vs.-S3. An additional analysis showed that 13,580 and 14,676 unigenes were up-regulated in S1-vs.-S2 and S1-vs.-S3, respectively, while 6981 and 9363 unigenes were down-regulated in these two groups, respectively (Figure 4c). ‘Plant hormone signal transduction’ were primarily enriched in either S1-vs.-S3 or S1-vs.-S2 group (Figure 4d and Figure S4a). The top regulatory pathways in the overlap part of S1-vs.-S2 and S1-vs.-S3 were ‘plant hormone signal transduction,’ ‘carbon metabolism,’ and ‘phenylpropanoid biosynthesis’ (Figure S4c). GO analysis showed that ‘nucleus’ and ‘DNA binding’ were the most enriched in the S1-vs.-S3 group (Figure 4e), while ‘plasma membrane’ and ‘oxidoreductase activity’ were enriched in the S1-vs.-S2 group and in the overlap part (Figure S4b,d). Taken together, 224 DEGs related to SE, cell division, cell fate specification (Table 3), and the regulatory pathways (Table S3), including hormones, epigenetic reprogramming, and stress responses, were identified based on the additional analysis of DEGs.

### 2.4. Identification and Analysis of the Profile of Expression of Putative Decisive TFs Associated with SE

**Figure 5:**
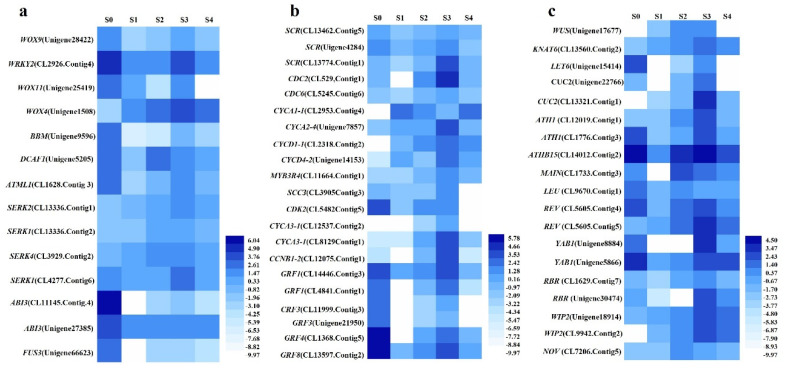
Identification and analysis of the DEGs annotated to somatic embryogenesis. (**a**) Putative decisive DEGs annotated to SE. (**b**) Important DEGs involved in cell division and expansion. (**c**) DEGs related with cell fate specification and shoot apical meristem (SAM) construction.

Decisive TFs that were annotated to SE, including *WUSCHEL-related homeobox 9* (*WOX9*, Unigene28422_All), *WOX11* (Unigene25819_All), *WRKY transcription factor 2* (*WRKY2*, CL2926.Contig4_All), *WOX4* (Unigene1508_All), *SERKs* (*SERK2*, CL13336.Contig1_All; *SERK1*, CL13336.Contig2_All and CL4277.Contig6_All; *SERK4*, CL3929.Contig2_All), *BBM* (Unigene9596_All), *AB13* (CL11145.Contig4_All and Unigene27385_All), *FUS3* (Unigene66623_All), *DDB1*- and *CUL4-associated factor homolog 1* (*DCAF1*, Unigene5205_All), and homeobox-leucine zipper protein *MERISTEM L1* (*ATML1*, Unigene10553_All and Unigene10553_All), were identified. The levels of expression of *WOX9* (Unigene28422_All), *WRKY2* (CL2926.Contig4_All), and *WOX11* (Unigene25819_All) were notably high in the zygotic embryo explant (S0), but they decreased substantially in S1, increased from S1 to S3, and then decreased in S4 (Figure 5a). The level of expression was the highest in the zygotic embryo explant (S0), followed by the somatic embryos in S3. The level of expression of *WOX4* (Unigene1508_All) increased from S0 to S3 and decreased in S4 with a significantly higher level in S3 than in the other stages. The levels of expression of three *SERKs* (*SERK2*, CL13336.Contig1_All; *SERK1,* CL13336.Contig2_Al; and *SERK4*, CL3929.Contig2_All) were enhanced in all processes (S1–S4) of SE, particularly in S3. They increased from S0 to S3 and decreased in S4 with a peak in the S3 of SE. The level of expression of another *SERK1* (CL4277.Contig6_All) decreased slightly in S1 and S2, increased dramatically in S3, and then decreased in S4 with the highest level in S3. The level of expression of *BBM* (Unigene9596_All) was notably high in the zygotic embryo explant (S0). There was no expression in S1, and the level of expression increased steadily in S2, climbed dramatically in S3, and then was substantially reduced greatly in S4. The levels of expression of *AB13* (CL11145.Contig4_All and Unigene27385_All) and *FUS3* (Unigene66623_All) were the highest in the zygotic embryo explant (S0). They decreased dramatically in S1, increased steadily in S2 and S3, and finally decreased again in S4. The level of expression of *DCAF1* (Unigene5205_All) was the highest in zygotic embryo explant. It decreased in S1, increased substantially in S2, and then decreased again in the latter two stages. The level of expression of *ATML1* (Unigene10553_All and Unigene10553_All) increased from S1 to S3 and then decreased in S4 with its greatest level in S3.

### 2.5. Identification and Analysis of the Profiles of Expression of Putative DEGs Annotated to Cell Division, Cell Fate Determination, and SAM Construction in SE

A total of 32 DEGs were annotated to cell division. Three DEGs were involved in asymmetric cell division, including *SCARECROW* (*SCR*, CL13462.Contig5_All and Unigene4284_All) and *SHORT-ROOT* (*SHR*, CL13774.Contig1_All). The levels of expression were all relatively high in S0, decreased in S1, increased from S1 to S3, and then decreased in S4 (Figure 5b). The level of expression of *SCR* was the highest in S0, followed by S3, while the level of expression of *SHR* was the highest in S3. There were 12 DEGs involved in cell cycle and division, including cell division control protein (*CDC2*, CL529.Contig1_All; *CDC6*, CL5245.Contig6_All), *CYCA1-1* (CL2953.Contig4_All), *CYCA2-4* (Unigene7857_All), *Cyclin-D1-1* (*CYCD1-1*, CL2318.Contig2_All), *CYCD4-2* (Unigene22536_All), *MYB3R-1* (CL11664.Contig1_All), *sister-chromatid cohesion protein 3* (*SCC3*, CL3905.Contig3_All), *Cyclin-dependent kinase G-2* (*CDKG-2*, CL5482.Contig5_All), *cyclin-A3-1* (*CYCA3-1*, CL12537.Contig2_All and CL8129.Contig1_All), and *G2/mitotic-specific cyclin S13-6* (*CCNB1-2*, CL12075.Contig1_All). All the DEGs related to cell division were substantially enhanced in S3. Six DEGs were associated with cell expansion, including *growth-regulating factor 1* (*GRF1*, CL14446.Contig3_All and CL4841.Contig1_All), *GRF3* (CL11999.Contig3_All and Unigene21950_All), GRF4 (CL1368.Contig5_All), and *GRF8* (CL13597.Contig2_All), and most of the level of expression decreased in S1, increased from S1 to S3, and then decreased in S4 with a relatively high level in the zygotic embryo explant (S0) and the S3 of SE.

Five DEGs were involved in the specification of cell fates, including *YABBY1* (*YAB1*, Unigene8884_All and Unigene5866_All), *Retinoblastoma-related protein* (*RBR*, CL1629.Contig7_All, and Unigene30474_All) and *Zinc finger protein* (*WIP2*, Unigene18914_All and CL9942.Contig2_All), and *NO VEIN* (*NOV*, CL7208.Contig5_All). The level of expression of *YAB1* decreased in S1 and increased consistently from S1 to S3 before decreasing in S4 (Figure 5c). The level of expression of one *YAB1* (Unigene8884_All) was the highest in S3 of SE, while that of the other *YAB1* (Unigene5866_All) was the highest in zygotic embryo explant (S0), followed by the S3 of SE. The level of expression of *RBR* (CL1629.Contig7_All and Unigene30474_All) also decreased from S0 to S1, increased from S1 to S3, and then decreased in S4 with its highest level in S3 of SE. The level of expression of *WIP2* (Unigene18914_All and CL9942.Contig2_All) increased from S0 to S3 and decreased in S4 with a peak in S3 of SE. The level of expression of *NOV* increased from S0 to S2 and then decreased in the latter two stages with its highest level in S2, followed by the S3 of SE.

**Figure 6:**
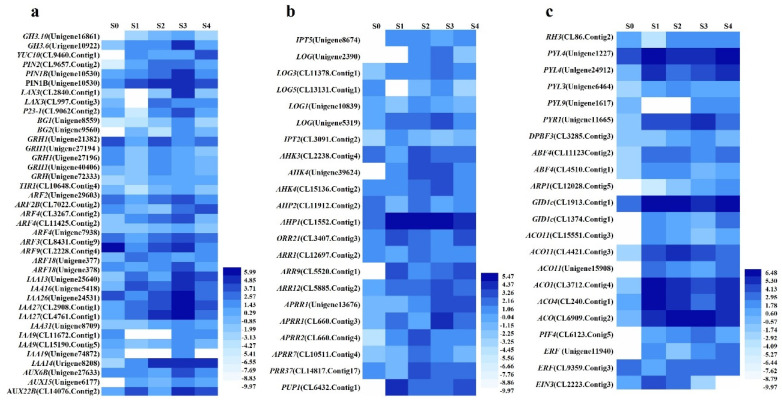
Identification and analysis of the DEGs annotated to the synthesis and signal pathways of hormones. (**a**) Auxin pathway. (**b**) Cytokinin pathway. (**c**) Pathways of other hormones, such as abscisic acid (ABA), gibberellic acid (GA), and ethylene.

Ten DEGs were related to meristem construction, including *WUS* (Unigene17677_All), homeobox protein *knotted-1-like LET6* (Unigene15414_All), Homeobox protein *knotted-1-like 6* (*KNAT6*, CL13560.Contig2_All), *CUC2* (Unigene22766_All and CL13321.Contig1_All), Homeobox protein *ATH1* (CL1776.Contig3_All), *MAINTENANCE OF MERISTEMS* (*MAIN*, CL1733.Contig3_All), Transcriptional corepressor *LEUNIG* (*LEU*, Unigene64502_All and CL9670.Contig1_All), and *REVOLUTA* (*REV*, CL5605.Contig5_All). All the DEGs involved in de novo meristem construction were significantly enhanced in the S3 stage (Figure 6c). The levels of expression of *WUS* (Unigene17677_All), *KNAT6* (CL13560.Contig2_All), one *CUC2* (CL13321.Contig1_All), *ATH1* (CL1776.Contig3_All) and *REV* (CL5605.Contig5_All) increased from the S0 to S3 stages and decreased in S4 with their highest level in S3. The levels of expression of *LET6* (Unigene15414_All) and another *CUC2* (Unigene22766_All) were notably high in the zygotic embryo explant (S0), and there was no expression in the normal callus in S1. It increased substantially from S1 to S3 and then decreased in S4 with a peak in S3. The level of expression of *LEU* (CL9670.Contig1_All) decreased in S1, increased in S2, and decreased in S3 and S4. There was no expression of *MAIN* (CL1733.Contig3_All) in S1. However, it increased dramatically to its highest level in S2 and then decreased from S2 to S4.

### 2.6. Identification and Analysis of the Profiles of Expression of Important DEGs Associated with Hormone Synthesis and the Signaling Pathway

A total of 85 DEGs annotated to hormone pathways including 38 DEGs annotated to the auxin pathway, 25 DEGs annotated to the cytokinin pathway, 10 DEGs annotated to the abscisic acid (ABA) pathway, 2 DEGs annotated to the gibberellin (GA) pathway, and 10 DEGs annotated to the ethylene pathway (Figure 6). Three DEGs were identified that were involved in the synthesis of auxin. The levels of expression of two *indole-3-acetic acid-amido* (*IAA*) *synthetase GH3.10* (Unigene16861_All) and *GH3.6* (Unigene10922_All) increased from S0 to S3 and decreased in S4 with a peak in S3 (Figure 6a). The level of expression of *indole-3-pyruvate monooxygenase YUC* (CL9460.Contig1_All) increased dramatically in S1, decreased in S2 and S3, and then increased in S4 with a relatively high level in S1 and S4. Nine DEGs were involved in auxin transporter or distribution. The levels of expression of the *auxin efflux carrier component* (*PINs*, CL9657.Contig2_All and Unigene10530_All) and one *BIG GRAIN 1* (*BG1*, (Unigene9560_All) increased from S0 to S3 and decreased in S4 with a peak in S3. The level of expression of the other *BG1* (Unigene8559_All) decreased in S1, increased from S1 to S3, and then decreased in S4 with a peak in S3. The level of expression of one *auxin transporter-like protein 3* (*LAX3*, CL2840.Contig1_All) increased from S0 to S2 and decreased in S3–4, and the other *LAX3* was highly enhanced in S3 to levels significantly higher than those of the other stages. The level of expression of *Co-chaperone protein p23-1* (*P23-1*, CL9062.Contig2_All) decreased in S1, increased to the highest level in S2, and then decreased in S3–4. There were six auxin receptors, including five *AUXIN SIGNALING F-BOX 2* (*AFB2*) and one *TRANSPORT INHIBITOR RESPONSE 1* (*TIR1*). Four *AFB2* (Unigene27194_All, Unigene27196_All, Unigene40406_All, and Unigene72333_All) and *TIR1* (CL10648.Contig4_All) were all enhanced in S2. They decreased first in S1, and substantially increased in S2, and then decreased in the latter two stages. The level of expression of another *AFB2* (Unigene21382_All) increased from S0 to S3 and decreased in S4 with a peak in S3. There were 22 *auxin-responsive proteins* (*ARFs*). Most of these *ARFs* were enhanced in S3. The level of expression of one *ARF4* (CL11425.Contig2_All) and *ARF5* (CL565.Contig2_All) increased from S0 to S3 and then decreased in S4 with a peak in S3. The levels of expression of *ARF3* (CL8431.Contig9_All), *ARF2* (Unigene29603_All), *ARF2B* (CL7022.Contig2_All), two *ARF4* (Unigene7938_All and CL3267.Contig2_All), and *ARF9* (CL2228.Contig4_All) decreased in S1, increased from S1 to S3, and then decreased in S4. The level of expression of one *ARF18* (Unigene377_All) increased in S1, decreased in S2 and S3, and then increased in S4 with its highest level in S1, while the level of expression of the other *ARF18* (Unigene378_All) decreased slightly in S1, increased from S1 to S3, and then decreased in S4. Moreover, the level of expression of all *IAAs* was enhanced in S3 with a significantly higher level than that of the other stages. Three auxin-induced proteins were identified. The levels of expression of *AUX6B* (Unigene27633_All), *AUX15* (Unigene6177_All), and *AUX22B* (CL14076.Contig2_All) were the highest in S3 with a similar trend.

Seven DEGs for cytokinin synthesis were identified. The level of expression of tRNA *dimethylallyltransferase 2* (*IPT2*, CL3091.Contig2_All) was enhanced in S1, decreased in S2 and S3, and then increased in S4 with a significantly higher level in S1 than that of the other stages (Figure 6b). The level of expression of *adenylate isopentenyltransferase 5* (*IPT5*, Unigene8674_All) increased from S0 to S2, decreased in S3, and then increased in S4 with a peak in S2. The level of expression of *cytokinin riboside 5′-monophosphate phosphoribohydrolase* (*LOG1*, Unigene10839_All) was also the highest in S2. The levels of expression of one *LOG* (Unigene2390_All) and *LOG3* (CL11378.Contig1_All) increased from S0 to S3 and decreased in S3 with a peak in S3. The level of expression of *LOG5* (CL13131.Contig1_All) decreased in S1, increased from S1 to S3, and then decreased in S4 with a peak in S3. The level of expression of the other *LOG* (Unigene5319_All) increased from S0 to S3, decreased in S2, and increased to its highest level in S3. A total of 14 cytokinin signal transduction genes were identified. The levels of expression of two *histidine kinase 3* (*AHK3*, CL2238.Contig4_All), *AHP2* (CL11912.Contig2_All), two-component *response regulator* (*ARR1*, CL12697.Contig2_All), and *PRR37* (CL14817.Contig17_All) decreased in S1, increased dramatically in S2, and then decreased in the latter two stages with a peak in S2. The levels of expression of *APRR2* (CL660.Contig4_All), *APRR7* (CL10511.Contig4_All), and one *AHK4* (Unigene39624_All) increased from S0 to S2 and decreased in S3 and S4 with their highest levels in S2, followed by S3. The levels of expression of the other *AHK4* (CL15136.Contig2_All), *APRR1* (Unigene13676_All), and *ARR12* (CL5885.Contig2_All) increased from S0 to S3 and decreased in S4 with a peak in S3. The levels of expression of *APRR1* (CL660.Contig3_All) was also the most enhanced in S3. The level of expression of another *histidine-containing phosphotransfer protein 1* (*AHP1*, CL1552.Contig1_All) increased substantially in S1 and decreased in the following three stages with the highest level in S1. The levels of expression of *ORR21* (CL3407.Contig3_All), *ARR9* (CL5520.Contig1_All), and one *purine permease 1* (*PUP1*, CL6432.Contig1_All) were first enhanced in S1, then reduced in S2 and S3, and increased in S4 with a relatively high level in S1 and S4.

**Figure 7:**
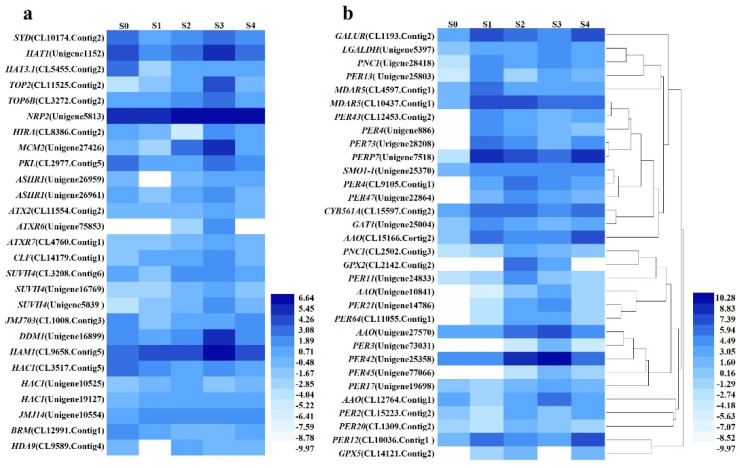
Identification and analysis of the DEGs annotated to the regulation pathways of epigenetic modifications and stress responses. (**a**) Heat maps describing the expression profiles of DEGs related to epigenetic modifications. (**b**) Heat maps describing the expression profiles of DEGs related to stress responses.

Seven DEGs were related to ethylene synthesis, including, *ACO4* (CL240.Contig1_All), *ACO* (CL6909.Contig2_All), and *PIF4* (CL6123.Contig5_All). The levels of expression of *1-aminocyclopropane-1-carboxylate oxidase 1* (*ACO1*, CL3712.Contig4_All) and *ACO4* (CL240.Contig1_All) were enhanced in S1; *ACO* (CL6909.Contig2_All) were enhanced in S2, while *PIF4* (CL6123.Contig5_All) was enhanced in S3 of SE (Figure 6c). Three DEGs were involved in signal pathway of ethylene. The levels of expression of *ethylene-responsive transcription factors* (*ERF*, Unigene11940_All) and *ethylene-insensitive protein3* (*EIN3*, CL2223.Contig3_All) were enhanced in S1, and the other *ERF* (CL9359.Contig3_All) was enhanced in S2 of SE. Therefore, most DEGs that annotated to the ethylene synthesis and signaling pathway were enhanced during the early stages (S1-S2) of SE. Moreover, two DEGs annotated to gibberellin receptor *GID1c* (CL1374.Contig1_All and CL1913.Contig1_All) were identified, and their levels of expression were highly enhanced during the whole process of SE (Figure 7a). Ten DEGs were annotated to ABA pathway. The levels of expression of *DEAD-box ATP-dependent RNA helicase 3 (RH3*, CL86.Contig2_All) decreased first and then increased with a relatively high level in S0 and S2–4 (Figure 7b). The levels of expression of *ABSCISIC ACID-INSENSITIVE 5-like proteins* (*DPBF3*, CL3285.Contig3_All) and one *abscisic acid receptor PYR1* (Unigene11665_All) shared a similar trend with a peak in S3. *ABF4* (CL4510.Contig1_All) and *abscisic acid receptor PYL3* (Unigene6464_All) were enhanced in S2. The levels of expression of *PYL4* (Unigene1227_All and Unigene24912_All) and another *ABF4* (CL11123.Contig2_All) were enhanced in S1 and S4. The levels of expression of *RNA-binding protein ARP1* (CL12028.Contig5_All) was the highest in S4, while that of *PYL9* (Unigene1617_All) was much higher in both S3 and S4. Thus, the levels of expression of the DEGs that annotated to the ABA synthesis and signaling pathway were enhanced during the developmental process of somatic embryos.

### 2.7. Analysis of the Profiles of Expression of Important DEGs Annotated to Epigenetic Modifications

A total of 27 DEGs were classified to epigenetic reprogramming. Most of epigenetic reprogramming DEGs, including *SYD* (CL10174.Contig2_All), *HAT1* (Unigene1152_All), *HAT3.1* (CL5455.Contig2_All), *TOP2* (CL11525.Contig2_All), *TOP6B* (CL3272.Contig2_All), *MCM2* (Unigene27426_All), *PKL* (CL2977.Contig5_All), *ASHR1* (Unigene26959_All and Unigene26961_All; *ATX2*, CL11554.Contig2_All; *ATXR6*, Unigene75853_All; *ATXR7*, CL4760.Contig1_All; CLF, CL14179.Contig1_All; *SUVH4*, CL3208.Contig6_All, Unigene16769_All, and Unigene5039_All), *JMJ703* (CL1008.Contig3_All), *DDM1* (Unigene16899_All), *HAM1* (CL9658.Contig5_All), and one *HAC1* (CL3517.Contig5_All) were enhanced in S3 of SE process (Figure 7a). The levels of expression of the other two *HAC1* (Unigene10525_All and Unigene19127_All) increased from S0 to S2 and then decreased in S3 and S4 with a peak in S2. The levels of expression of the negative regulators *JMJ14* (Unigene10554_All), *BRM* (CL12991.Contig1_All) and *HDA9* (CL9589.Contig4_All) were substantially greatly reduced in S3.

### 2.8. Identification and Analysis of the Profiles of Important DEGs Associated with Stress Responses

A total of 32 DEGs were annotated to antioxidant molecules and enzymes, including one *D-galacturonate reductase* (*GALUR*, CL1193.Contig2_All), one *L-galactose dehydrogenase* (*LGALDH*, Unigene5397_All), two *monodehydroascorbate reductase* (*MDAR5*, CL10437.Contig1_All and CL4597.Contig1_All), four *L-ascorbate oxidase* (*AAO*, CL12764.Contig1_All, Unigene27570_All, CL15166.Contig2_All, and Unigene10841_All), one *methylsterol monooxygenase 1-1* (*SMO1-1*, Unigene25370_All), one *cationic peroxidase 1* (*PNC1*, CL2502.Contig3_All and Unigene28418_All), 16 *peroxidases* (*PER11*, Unigene24833_All; *PER12*, CL10036.Contig1_All; *PER13*, Unigene25803_All; PER17, Unigene19698_All; *PER2*, CL15223.Contig2_All; *PER20*, CL1309.Contig2_All; *PER21*, Unigene14786_All; *PER3*, Unigene73031_All; *PER4*, CL9105.Contig1_All and Unigene886_All; *PER42*, Unigene25358_All; *PER43*, CL12453.Contig2_All; *PER45*, Unigene77066_All; *PER47*, Unigene22864_All; *PER64*, CL11055.Contig1_All; *PER73*, Unigene28208_All), one *peroxidase P7* (*PERP7*, Unigene7518_All), two *glutathione peroxidase 2* (*GPX2*, CL2142.Contig2_All; *GPX5*, CL14121.Contig2_All), one *transmembrane ascorbate ferrireductase 1* (CYB561A, CL15597.Contig2_All), and one *Thioredoxin M3* (*GAT1*, Unigene25004_All). A total of 16 DEGs annotated to stress responses were enhanced in S1, and 15 DEGs were the highest in S2 and S3 with a trend of first increasing and then decreasing (Figure 7b).

### 2.9. Validation of the Expression of Important Candidate DEGs by qRT-PCR

**Figure 8:**
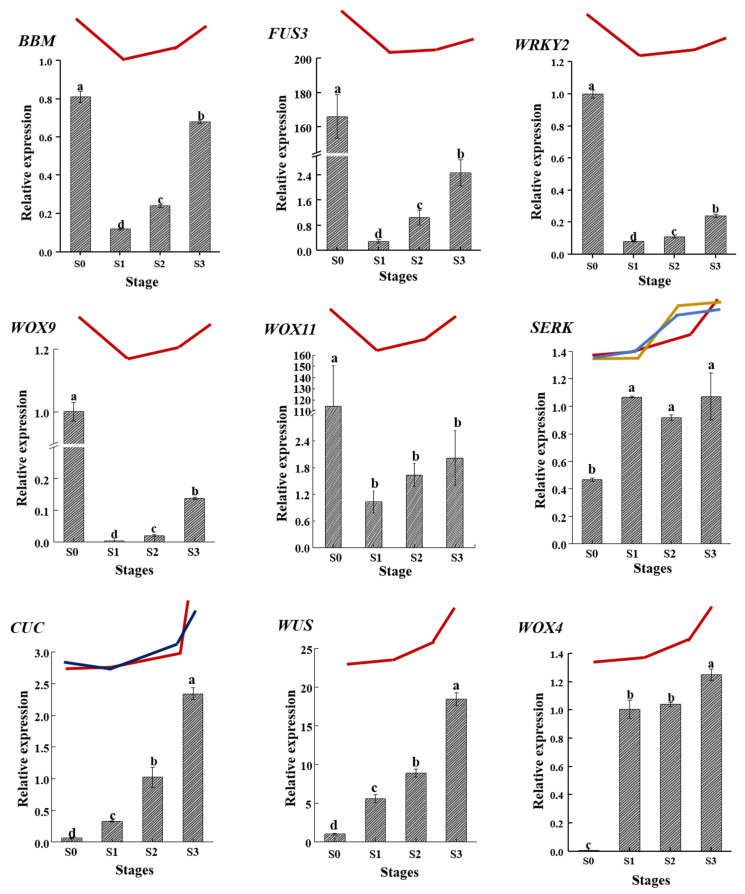
Validation of the expression of nine important candidate DEGs by qRT-PCR. Different lowercase letters indicate significant differences among different stages (Duncan’s test at *p* < 0.05 after analysis of variance). The broken line represents the expression patterns detected by transcriptome analysis. There were three *SERKs* and two *CUC* detected by transcriptome analysis. The patterns of expression were shown in different colors.

To confirm the accuracy of the high-throughput sequencing results, nine important DEGs involved in SE were analyzed by qRT-PCR. The results showed that all nine DEGs were generally consistent with the RNA-Seq datasets. The levels of expression of *BBM*, *FUS3*, *WRKY2*, *WOX9*, and *WOX11* were the highest in the zygotic embryo explant (S0), decreased substantially in S1 and gradually increased from S1 to S3, with the highest level in S3 of the SE. The levels of expression of *SERK*, *CUC*, *WUS*, and *WOX4* increased from S0 to S3 with a significantly higher level in S3 than in the other stages (Figure 8).

## 3. Discussion

**Figure 9:**
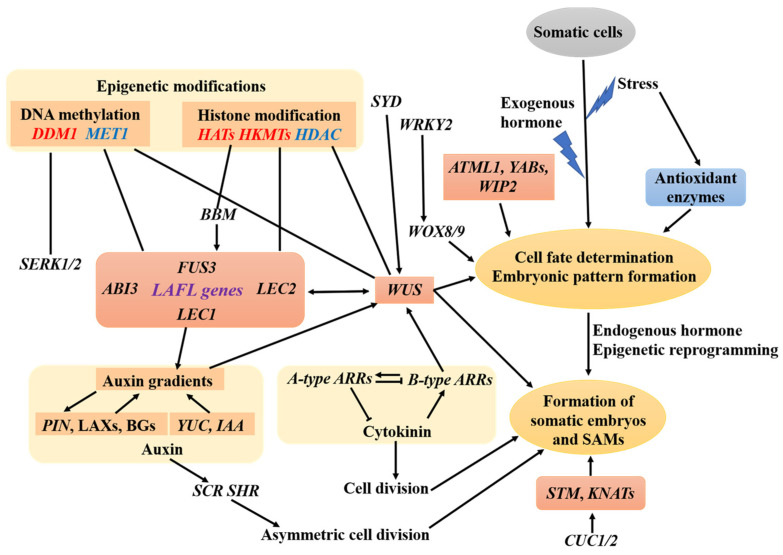
A model of a molecular regulatory network during tree peony somatic embryogenesis. Black arrows indicate positive regulation. Black lines without arrows mean either positive or negative regulation. Genes marked red related to epigenetic modification accelerate somatic embryogenesis, while genes marked in blue inhibit somatic embryogenesis.

Plant regeneration via SE could highly increase the speed of propagation and thereby shorten the breeding period. Plant regeneration from tree peony zygotic embryos has been studied in recent decades, and some progress has been made. However, the regenerative capacity was very low and depended substantially on the genotypes [13]. The inner molecular mechanisms were still unclear. In this study, we established an effective protocol for a plant regeneration system in tree peony via SE using the zygotic embryo as the explant. Based on this efficient plant regeneration system, we analyzed the molecular dynamics during SE. The important genes and probable pathways that regulate shoot regeneration via SE were identified in tree peony. KEGG and GO analyses demonstrated that the DEGs annotated to hormone pathways, particularly the cytokinin and auxin pathways; epigenetic modifications, stress responses, cell division, cell fate determination, meristem construction, and SE-specific genes were significantly enriched, which is consistent with previous studies [32,33] indicating that SE and the enhanced regenerative capacity are associated with the related processes described above. Accordingly, the most frequently represented TFs during tree peony somatic embryogenesis were *ARFs*, *MYB*, *bHLH*, *ARR-B*, *AP2-EREBP*, *NAC*, *GRAS*, *ABI3VP1*, *WRKY*, and *bZIP*, which were substantially up-regulated in embryonic callus (S2) and somatic embryos (S3) compared with that of the non-embryonic callus (S1), indicating that these TFs are closely related to embryonic transition and somatic embryo formation. Similar TFs have also been detected in maize through transcriptome analysis [34]. *ARFs* are important auxin response factors, and *B-type ARRs* are important cytokinin response regulators. Both play vital roles in SE [19,35,36] and demonstrate the important roles of auxin and cytokinin in the regulation of SE. It has also been reported that auxin and stress often work in concert to modulate SE [37,38]. *MYB*, *bHLH*, *NAC* (including *CUC2*), *WRKY*, *bZIP*, *GRAS*, and *ABI3VP1* (such as *ABI3*, *FUS3*, *LEC2*) are involved in stress responses and may crosstalk with hormones to regulate SE [34,39,40,41,42]. In addition, hormones, particularly auxin and cytokinin, could alter epigenetic modifications and induce SE [9]. Therefore, hormones, particularly auxin and cytokinin, could highly function in concert with stress responses and epigenetic modifications to control the induction of SE and development of somatic embryos. Based on the expression patterns in our study and according to the prior research [43,44,45,46,47], we proposed a model of a molecular regulatory network during tree peony SE (Figure 9).

### 3.1. Establishment of an Efficient Plant Regeneration System via SE in Tree Peony

SE is a powerful tool that is widely used for commercial plant propagation and breeding by genetic engineering. This study showed that MS medium supplemented with 3.0 mg L^−1^ 6-BA and 1.0 mg L^−1^ NAA could successfully induce embryonic callus and SE in tree peony. WPM medium supplemented with 1.0 mg L^−1^ 6-BA and 0.5 mg L^−1^ GA_3_ (SI3) enhanced the development of shoots from somatic embryos, and WPM medium supplemented with 3.0 mg L^−1^ BA, 2.0 mg L^−1^ IBA, and 1.0 mg L^−1^ acetylsalicylic acid in concert with a 15-day period of cold treatment accelerated the induction of roots. The whole success induction ratio from S0 to S4 was 55.56%. Progress has also been made on plant regeneration from zygotic embryo explants of tree peony in our previous research and that of Du et al. (2020) with an embryo induction ratio of 48% [10,12]. Therefore, the induction of embryonic callus and the development of somatic embryos was much enhanced in this study compared with the previous studies. Based on the established plant regeneration system, the underlying molecular mechanisms of SE were further analyzed in more detail in tree peony using transcriptome sequencing techniques.

### 3.2. Analysis of the Putative Decisive TFs Annotated to SE

*WOX9* is important in the initiation of SE, and its overexpression leads to an increase in embryogenic capacity in *Medicago truncatula* [48]. *WRKY2* regulates the patterns of division of the basal cells during the early stage of embryogenesis by activating the expression of *WOX8* and *WOX9* [44]. *WOX11* promotes cell fate specification during embryogenesis [49]. *WOX4* accelerates embryo development and germination during SE in grape [50]. In this study, the levels of expression of *WOX9*, *WRKY2*, and *WOX11* were the highest in zygotic embryo explants (S0). They were reduced in S1 and highly enhanced in the somatic embryos (S3). *WOX9* is also highly expressed in the somatic embryos of Norway spruce (*Picea abies*) and *M. truncatula* [51,52]. The level of expression of *WOX4* was enhanced in all stages (S1–S4) of SE, and it was the highest in somatic embryos (S3). Similar results were observed in *V. vinifera* [50]. *SERK* plays a key role in embryogenic competence acquisition and SE [53]. The level of expression of *SERK* was enhanced in all processes (S1–S4) of SE, particularly in the somatic embryo formation (S3). *SERK1* and *SERK2* are highly expressed during embryogenic formation and the developmental stages in *A. thaliana* and *Oryza sativa*, respectively [54,55]. The *LEC1-ABSCISIC ACID INSENSITIVE 3* (*ABI3*)-*FUS3-LEC2 (LAFL*) complex and *BBM* are master regulators of SE, and *BBM* activates the *LAFL* network [56]. The ectopic expression of *BBM* triggers a conversion from vegetative to embryonic growth [57]. In this study, high expressions of *BBM, AB13* and *FUS3* were identified in zygotic embryo explants (S0) and in somatic embryos (S3). In conifers, the levels of expression of *SERK* and *BBM* increase during the later development of SE [58]. *DCAF1* is essential for plant embryogenesis [59], while *ATML1* plays an important role in embryonic pattern formation [60]. The levels of expression of *DCAF1* were enhanced in embryonic callus (S2), while the level of expression of *ATML1* was the highest in somatic embryo (S3). The high levels of expression of the above DEGs in the somatic embryos (S3) indicates their decisive roles in SE.

### 3.3. Identification and Analysis of the Profiles of Expression of Putative DEGs Annotated to Cell Division, Cell Fate Determination and De Novo Meristem Construction Involved in the Process of SE

Plant regeneration is accompanied by the establishment of new stem cells and meristems, which often require the reactivation of potential cell division [61]. Asymmetric cell divisions are central to the establishment of SAM and root apical meristem (RAM), determination of cell fate, and development of embryos [62]. In embryogenesis, asymmetric cell division forms a spherical proembryo, and the SAM is generated from the four most apical cells [63]. *SCR* and *SHR* are involved in asymmetric cell division [64,65]. We found that *SCR* and *SHR* were enhanced in the somatic embryo formation process (S3). Thus, all the DEGs related to cell division and expansion were significantly enhanced in this stage. Similar results were found in alfalfa (*Medicago sativa*) [66]. These results showed that there were active asymmetric divisions and normal cell division during somatic embryo formation (S3), which substantially contributes to the establishment of SAM.

The de novo construction of SAM is essential for shoot regeneration by either organogenesis or SE, and the acquisition of stem cell identity is extremely important for the de novo construction of SAM [18]. SAM is always established during somatic embryo formation in embryogenesis [63]. *YABs* are involved in the determination of abaxial cell fate and regulate the initiation of embryonic SAM development during embryogenesis [45]. *RBR* plays a central role in controlling the cell fate establishment and meristem cell differentiation during the process of plant regeneration [67,68]. *NOV* is essential for the determination of cell fate during embryogenesis [69], and *WIP2* is involved in determination of stem cell fate within embryonic meristems [46,70]. In this study, the levels of expression of *YABs*, *RBR*, and *WIP2* were enhanced in the somatic embryo formation process (S3), and the level of expression of *NOV* was the highest in embryonic callus (S2). *WUS*, a transcription factor expressed in the organizing center of SAM, determines the fate of stem cells. It interacts with *CLV* to produce a *WUS/CLV* self-regulatory loop that is critical for the maintenance of stem cell identity in SAM during SE [71]. *Class I KNOX* genes, including *KNAT1* and *KNAT6*, are required for the initiation and maintenance of SAM during embryonic development [72,73]. *CUC1* and *CUC2* are involved in regulating the formation of SAM during embryogenesis by activating *STM* and *KNAT6* [72]. Other important TFs, including *ATH1*, *ATHBs*, *REV*, *MAIN*, and *LEU*, are also involved in the regulation of the initiation, maintenance, and development of SAM [74,75,76,77]. In this study, the levels of expression of *WUS*, *KNAT6*, *CUC2*, *ATH1*, *ATHB15*, and *REV* were all substantially enhanced in the somatic embryos (S3). The level of expression of *MAIN* was the highest in embryonic callus (S2), followed by somatic embryos (S3). One *LEU* (Unigene64502_All) was expressed at the highest level in somatic embryos (S3), while the other *LEU* (CL9670.Contig1_All) was enhanced in embryonic callus (S2). Similarly, a high level of expression of *WUS* was also detected in the embryonic callus and somatic embryos in *A. thaliana* [47]. The levels of expression of *KNAT6* and *CUC* are elevated during embryogenesis, so that SAM is established [72]. Therefore, most of the DEGs related to the construction of SAM were enhanced in somatic embryo formation process (S3), proving that SAM was established during the process of somatic embryo formation (S3), and the DEGs described were important participants in this process. In addition, the trends of expression of nine decisive DEGs in SE detected by qRT-PCR were generally consistent with the RNA-Seq datasets. Therefore, the most decisive and most frequently represented genes, including *SERK*, *WOX9*, *WOX11*, *WOX4*, *WRKY2*, *BBM*, *FUS3*, *CUC*, and *WUS*, were characterized as the molecular markers for tree peony SE.

### 3.4. Identification and Analysis of the Profiles of Expression of the Important DEGs Annotated to Hormone Pathways and Their Roles in the Regulation of SE

Plant growth regulators play vital roles in SE [78]. Auxin promotes the acquisition of cell totipotency and induces SE by altering the accessibility of chromatin [15]. Auxin biosynthesis maintains embryo identity and growth during SE [79]. We found that *YUC* was enhanced in S1 and S4, while the IAA synthetase *GHs* were increased in S3 of the SE process, indicating the important role of auxin synthesis in the induction of callus, the construction of SAM, and the formation and development of embryos in tree peony. The establishment of auxin gradients correlates with the expression of *WUS* and activates the polar localization of *PIN1*, which gives rise to the formation of SAM during SE [47,80]. *PINs*, *LAXs*, and *BG1* regulate polar auxin transport [81] and play a regulatory role in SE [82]. In this study, the levels of expression of *PINs* and *BG1* were enhanced in S3. The levels of expression of two *LAX3* were enhanced in S2 and S3, respectively. The *PIN1* is substantially enhanced during the specific place of *A. thaliana*, which is critical for the regulation of *WUS* during SE [47]. The results showed that polar auxin transport genes function concomitantly with the auxin synthesis genes to regulate the establishment of auxin gradients in stages S2–S3 of SE in tree peony. Auxin synthesis, transport, and signal pathway genes are also up-regulated and have been identified in maize (*Zea mays*) during SE [83]. *AFB2*, one auxin receptor, is involved in auxin-regulated embryogenesis [84]. The levels of expression of *AFB2* and the other DEGs associated with auxin receptors were enhanced in the embryonic callus (S2), indicating that a receptor perceived auxin as a signal during this stage. *ARFs* and *IAAs* play key roles in regulating auxin-responsive transcription [85]. In *Arabidopsis*, multiple *arf* mutants displayed SE defects [35,36]. *IAA9* has been shown to be upregulated during the initiation of SE [86]. Most of the DEGs of *ARFs* and all those of the *IAAs* were enhanced in somatic embryos (S3), which was similar to the results observed in rubber tree (*Hevea brasiliensis*) [87], proving again that auxin has a critical role in the regulation of SE.

The determination of cell fate and the de novo construction of SAM are primarily regulated by auxin and cytokinin [88,89]. It has been reported that a deficiency of cytokinin reduced the size and activity of SAM [90]. In this study, *cytokinin synthesis IPT2* was enhanced in S1; *IPT5* and one *LOG1* were enhanced in S2; while the other four *LOGs* were enhanced in S3, indicating that *cytokinin* plays important roles in the process of SE. *AHKs* are cytokinin receptors, and *AHPs* act as signaling shuttles between the nucleus and cytoplasm [21]. The cytokinin response is mediated by *B-type ARRs* as positive regulators in the two-component cytokinin signaling pathway, whereas *type-A ARRs* function as negative regulators of the downstream responses [91]. In *A. thaliana*, *B-type ARRs* (*ARR1*, *ARR2*, *ARR10*, and *ARR12*) play essential roles in the de novo construction of SAM [19]. In addition, cytokinin can modulate the accessibility of chromatin and the expression of *WUS* through the *B-type ARRs* [11,21]. The genes involved in cytokinin biosynthesis and signal transduction pathways increase remarkably in expression during SE in cotton (*Gossypium* spp.) [92]. The levels of expression of *ARRs* are enhanced during the process of SAM establishment and maintenance in *A. thaliana* [21]. The levels of expression of the DEGs annotated to cytokinin signaling were also elevated during the process of SE. The level of expression of *AHP1* was the highest in S1. *AHK3*, *ARR1*, *APRR2*, *APRR7*, and *PRR37* were expressed at their highest levels in S2. *APRR1* and *ARR12* were the most enhanced in S3, which indicates the important roles of these cytokinin signaling genes in the SE of tree peony.

Ethylene, GA, and ABA are also involved in the induction or development of the somatic embryos [78]. Ethylene modulates the induction of somatic embryos and the development of globular embryos [93], which is markedly connected to hormonal crosstalk (with auxin and cytokinin in particular), as well as stress responses [94]. Most of the DEGs that were annotated to the ethylene synthesis and signaling pathway were enhanced during the early stages (S1–S2) of SE, indicating their roles in the induction of SE. GA_3_ enhances the level of expression of a *KNOTTED-like homeobox* gene (*KNOX*) and stimulates the formation and germination of somatic embryos [95]. We also found that the level of expression of *GID1c* was substantially enhanced during the whole process of SE. ABA induces SE and is essential for the acquisition of embryogenic competence [96,97]. In this study, the ABA biosynthetic gene was expressed at its highest levels in S2 with a relatively high level in S2–S4. Thus, the levels of expression of DEGs annotated to the ABA signal pathway were enhanced in the late stages of SE. ABA biosynthesis, receptors and signaling response genes were also up-regulated during the SE of *A. thaliana* [98]. Therefore, ethylene, GA, and ABA also play important roles in the induction and development of SE in tree peony.

### 3.5. Identification and Analysis of the Profiles of Expression of the Important DEGs Annotated to the Regulation of Epigenetic Modifications and Their Roles in the Regulation of SE

Epigenetic modifications include DNA methylation and histone modifications, such as acetylation, methylation, and phosphorylation, that orchestrate the structure and accessibility of chromatin and regulate global reprogramming of the cell transcriptome [99]. In recent years, epigenetic modifications have emerged as critical factors for the control of the transition of cell fate, callus formation, and SE through extensive transcriptome reprogramming [15,100]. DNA methylation is one of the most studied epigenetic mechanisms owning to its essential role in gene expression and SE [101]. The balance between hypermethylation and hypomethylation is key to the success of SE [102,103]. *DDM1* is required to maintain the DNA methylation status and promote chromatin remodeling [104]. The level of expression of DDM1 was up-regulated during SE, particularly in S3.

Histone modification is one of the most important epigenetic modifications, and it plays a key role in the regulation of gene expression [105]. *Histone acetyltransferases* (*HATs*) promote the open state of chromatin [106], while *histone deacetylases* (*HDACs*) trigger the condensation of chromatin [107]. These two antagonistically acting enzymes work in concert to control SE by regulating gene expression [99,108]. Histone deacetylase inhibitors have been reported to accelerate the expression of embryogenesis-related genes and increase embryogenic potential [109]. *Histone acetyltransferase1* (*HAC1*) acetylates histones, thus providing a specific tag for transcriptional activation and promoting the expression of *WUS* and SAM organization [110]. *Histone lysine methyltransferases* (*HKMTs*) are extremely important enzymes that modify chromatins, which transcriptionally activate or repress genes by regulating their chromosomal states [111]. H3K4, H3K36, and H3K79 methylations are involved in active transcription, while H3K9, H4K20, and H3K27 methylations are associated with gene silencing [112]. *Class III HKMTs*, including *ATX1-5*, regulate the establishment of shoot identity via H3K4 trimethylation and demethylation, while *ATXR7* may accelerate the demethylation of H3K36 [25]. Moreover, *SPLAYED* (*SYD*), a catalytic component of the chromatin structure-remodeling complex, controls the fate of stem cells via the regulation of promoter of *WUS* in the SAM [113]. The levels of expression of histone modification genes are highly elevated in the SE of rubber tree (*Hevea brasiliensis*) [87]. This study also showed that most of the epigenetic genes (78.6%) were highly enhanced in S3 of the SE process. The level of expression of *HAC1* peaked in S2, while the level of expression of negatively regulated genes, including JMJ14, *BRM*, and *HDA9*, were highly reduced in S3, indicating that epigenetic modifications, particularly those involved in histone modifications, played crucial roles in the regulation of SE.

Moreover, it has been widely reported that hormones, particularly auxin and cytokinin, could modify the levels of epigenetic modifications, which causes an extensive reprogramming of the transcriptome and finally contributes to SE [9]. Exogenous hormones such as auxin can modify the levels of DNA methylation in embryogenic cells, which regulates the expression of genes involved in SE, such as *BBM1*, *WUS*, and *LEC* [78]. The removal of H3K27me3 at the *WUS* locus is a prerequisite for its induction by the *cytokinin response factors B-type ARRs* [19]. In this study, the results also showed that the expression of part of the DEGs involved in auxin and cytokinin pathways share similar trends with epigenetic modification genes, and part of them peaked earlier than the genes for epigenetic modification, indicating that the effect of auxin and cytokinin precedes epigenetic modification or functions at the same time.

### 3.6. Analysis of the Important DEGs Annotated to Stress Responses and Their Relationship with SE

The ROS produced during stress condition is known to function as a signal that regulates plant growth and development [114]. Moreover, stress induces the remodeling of fate of plant cells and induces SE in concert with hormone-regulated pathways [115]. It has been reported that cellular stress conditions prompt vegetative cells to acquire embryogenic competence through cellular reprogramming [53]. A high content of catalase and ascorbate peroxidase has a stimulatory effect on SE [30]. Cationic peroxidase is required for SE in asparagus (*Asparagus officinalis*) [116]. The activities of peroxidase are always substantially enhanced in embryonic callus compared with non-embryonic calli. Therefore, peroxidases are used as markers of the capacities of SE in different species [31,117]. In addition, the ROS homeostasis in stress conditions often engages in crosstalk with the auxin pathways to induce SE [37]. In this study, 32 DEGs were annotated to antioxidant molecules and enzymes, and all of them were up-regulated in SE. This was similar to the trends of expression of the DEGs involved in auxin synthesis and signaling pathways. Peroxidase transcripts were also expressed differently at different stages of SE in wheat (*Triticum aestivum*) and oil palm (*Elaeis guineensis*) [118,119]. These results indicate that stress and auxin may work in concert to accelerate SE.

## 4. Materials and Methods

### 4.1. Plant Materials and Culture Conditions

*P. ostii* ‘Fengdan’ plants were cultured in the resource garden of the Institute of Vegetables and Flowers, Chinese Academy of Agricultural Sciences, Beijing, China. Uniformly sized full and glossy seeds were collected in September 2020 and washed three times in tap water with a few drops of detergent for 10 min, and then immersed in sterilized water for 24 h. The seeds were further disinfected using 3% sodium hypochlorite (NaClO) for 5 min, 75% ethanol for 3 min, and finally rinsed five times with sterilized water. Zygotic embryos were picked out from the sterilized seeds and cultured on Murashige and Skoog medium (MS) supplemented with 3.0 mg L^−1^ 6-benzylaminopurine (6-BA), 1.0 mg L^−1^ 1-naphthylacetic acid (NAA), 4.0 mg L^−1^ Phytagel, and 30 g L^−1^ sucrose under dark and poikilothermic (22 °C for 16 h and 18 °C for 8 h) conditions for 3 months to induce the callus and somatic embryos. Those regenerated somatic embryos were then cultured on shoot induction medium, including SI1 [(woody plant medium) WPM + 4.0 mg·L^−1^ phytagel + 30 g·L^−1^ sucrose + 0.5 mg·L^−1^ 6-BA + 0.5 mg·L^−1^ GA_3_], SI2 (WPM + 4.0 mg·L^−1^ phytagel + 30 g·L^−1^ sucrose + 0.5 mg·L^−1^ 6-BA + 1.0 mg·L^−1^ GA_3_), SI3 (WPM + 4.0 mg·L^−1^ phytagel + 30 g·L^−1^ sucrose + 1.0 mg·L^−1^ 6-BA + 0.5 mg·L^−1^ GA_3_), and SI4 (WPM + 4.0 mg·L^−1^ phytagel + 30 g·L^−1^ sucrose + 1.0 mg·L^−1^ 6-BA + 1.0 mg·L^−1^ GA_3_) for shoot development. When the shoots had grown to 3–4 cm, each was separated and cultured on root induction medium, RI1 (WPM + 4.0 mg·L^−1^ phytagel + 30 g·L^−1^ sucrose + 3.0 mg·L^−1^ 6-BA + 3.0 mg·L^−1^ IBA + 2.0 mg·L^−1^ caffeic acid), RI2 (WPM + 4.0 mg L^−1^ phytagel + 30 g·L^−1^ sucrose + 3.0 mg·L^−1^ 6-BA + 3.0 mg·L^−1^ IBA + 1.0 mg·L^−1^ acetylsalicylic acid), RI3 (WPM + 4.0 mg·L^−1^ phytagel + 30 g·L^−1^ sucrose + 3.0 mg·L^−1^ 6-BA + 2.0 mg·L^−1^ IBA + 1.0 mg·L^−1^ acetylsalicylic acid) at 4 °C for 15 days, followed by a poikilothermic (22 °C for 16 h and 18 °C for 8 h) condition for 45 days to induce roots. The ratio of explants with callus induction, embryonic callus induction, shoot induction, and root induction were recorded after 14 days, 2 months, 4 months, and 6 months of culture, respectively. The number of shoots per explant was also determined after 4 months of culture. Samples at different culture stages including S0 (zygotic embryo explant, ZE), S1 (non-embryonic callus, after 1 month of culture), S2 (embryogenic callus, after 2 months of culture), S3 (somatic embryos, after 3 months of culture), and S4 (the regenerated shoots after 4 months of culture) were also collected for histological and transcriptomic analyses. The former samples were stored in 2.5% glutaraldehyde and incubated at 4 °C for 8 h, while the latter samples were immediately frozen in liquid nitrogen and stored at −80 °C until use.

### 4.2. Morphology and Histological Analysis of Whole Plant Regeneration Process

All the induction stages were recorded and photographed using a Leica Stereo Microscope (Leica LED2500, Wetzlar, Germany). For scanning electron microscopy (SEM) observation, the samples in 2.0% glutaraldehyde were washed with 0.1 M phosphate buffer (PBS, pH 7.0) and fixed by 1% osmic acid (OsO_4_) for 2 h at 4 °C. Subsequently, they were dehydrated in a series of ethanol (30%, 50%, 70%, 90%, and 100%) and then dried, gold coated, and photographed with a SU-8010 (Hitachi, Tokyo, Japan) scanning electron microscope.

### 4.3. RNA-Seq, cDNA Library Construction, and Sequence Assembly and Annotation

Total RNA from samples at five different stages (S0–S4) was extracted using Trizol extraction kit (Invitrogen, CA, USA) according to the manufacturer’s instructions. Two embryos, calli or shoots were used in each sample. After digestion with RNase-free recombinant DNase I, the quality and quantity of the total RNA were determined by NanoDrop spectrophotometer (Thermo Scientific™, Waltham, MA, USA). High-quality RNA from the five samples were used for cDNA library construction and BGISEQ-500 RNA-Seq. The cDNA Library preparation was performed following the Illumina manufacturer’s instructions. The double-stranded cDNA of the above five samples was sequenced using an Illumina HiSeq™ 4000 (4 × 100 bp read length) platform at the Beijing Genomics Institute Company, Shenzhen, China. All transcription sequencing data raw files has been deposited at the NCBI Sequence Read Archive under the BioProject ID PRJNA864612.

The quality of raw reads from each library was assessed using the FASTQC program and Trimmomatic software (0.39) to filter the reads and remove adapter sequences. The clean reads were then assembled de novo using Trinity to obtain reference transcriptome unigenes for annotation and DGE analysis. The quality of the assembled transcripts was evaluated using the Benchmarking Universal Single-Copy Orthologs (BUSCO) database (https://busco.ezlab.org/, accessed on 1 August 2021). The longest open reading frame (ORF) per transcript contig was identified using TransDecoder. The sequence similarities and ORFs were predicted and identified by BLASTP, ORF finder, and TransDecoder software (v.5.1) (Created by Broad Institute, MA, USA). The Unigenes were annotated and functionally classified by conducting a BLAST search against seven databases, including NR (NCBI non-redundant protein sequences), NT (NCBI non-redundant nucleotide sequences), Pfam (the prediction of protein structure domain), Kyoto Encyclopedia of Genes and Genomes (KEGG), Eukaryotic Orthologous Groups of proteins/Clusters of Orthologous Groups of proteins (KOG/COG), KEGG Orthology (KO), and Gene Ontology (GO).

### 4.4. Analysis of Differentially Expressed Genes, and GO and KEGG Enrichment

The differentially expressed genes (DEGs) were analyzed using DESeq2. The false discovery rate (FDR) is used as a correction in many applied multiple testing problems. The parameters were set at *p*-adjust of < 0.05, false discovery rate (FDR) ≤ 0.001, and log2(Fold change) ≥2 with fragments per kilobase million (FPKM) of >1 in at least one sample. GO and KEGG enrichment analyses were performed using Fisher’s exact test for the elucidation of the biological functions of the genes. The heatmap of DEGs was constructed using Heml software (Version 1.0) (Created by Huazhong University of Science and Technology, Wuhan, China).

### 4.5. Validation of Gene Expression by Quantitative Real-Time PCR (qRT-PCR)

Important DEGs related to SE were selected to check their relative gene expression by qRT-PCR. RNA was extracted from the samples using a total RNA extraction kit (Tiangen, Beijing, China) according to the manufacturer’s protocol. The quality and quantity of RNA were assessed with 1.2% agarose electrophoresis and a NanoDrop 2000c spectrophotometer (Thermo Scientific, Waltham, MA, USA), respectively. Primers were designed using Premier 5.0 software (Created by Premier company, Toronto, Canada). The first-strand cDNA synthesis was performed using a FastQuant RT Kit (Tiangen, Beijing, China) following the manufacturer’s instructions. The relative expression of the candidate genes was calculated using a double standard curve according to the CFX96 Real-Time system (Bio-Rad, Hercules, CA, USA) by normalizing to the reference gene ACTIN according to Wang et al. (2020). All qRT-PCR reactions were performed in triplicate with at least three biological replicates. The primer information used in this experiment are shown in Table S4.

### 4.6. Data Analysis and Statistics

The experimental assays used to obtain all results were repeated at least three times. Results were presented as means ± the standard error (SE) and analyzed using one-way ANOVA, followed by Duncan’s multiple-range test. Statistical analysis was performed with SPSS 22.0 (SPSS Institute, IBM, Endicott, NY, USA). 

## 5. Conclusions

A highly efficient regeneration system of tree peony via SE was established. Based on this regeneration system, the transcriptomes of five SE stages were analyzed. Totals of 32,176, 20,561, and 24,039 DEGs were identified in pairwise comparisons of S0-vs.-S1, S1-vs.-S2, and S1-vs.-S3, respectively. Functional characterizations of the DEGs based on GO and KEGG analyses are presented. A total of 224 DEGs were identified for their potential associations with SE and the regulatory pathways, including 26 decisive DEGs in SE and SAM construction, 21 DEGs annotated to cell division and expansion, seven DEGs annotated to cell fate determination, 37 DEGs annotated to auxin signaling pathway, 24 DEGs annotated to cytokinin signal pathway, 10 DEGs annotated to the ABA signaling pathway, 2 DEGs annotated to the GA signaling pathway, and 10 DEGs annotated to the ethylene signaling pathway, 54 DEGs annotated to the central histones of nucleosome and epigenetic modifications, and 31 DEGs annotated to stress responses. The genes involved in these processes were discussed in this study, which helps to elucidate their roles in SE. The temporal program for gene expression by qRT-PCR during SE was also analyzed, and the results confirmed the patterns of gene expression of the transcriptomes. Taken together, cell division, particularly asymmetric cell division was enhanced during SE. Moreover, the determination of cell fate and the genes related to meristem formation played central roles in the construction of SAM during somatic embryo formation. The hormone signaling pathways work in concert with epigenetic modifications and stress responses to regulate the induction of SE and the development of somatic embryos. *SERK*, *WOX9*, *BBM*, *FUS3*, *CUC*, and *WUS* were characterized as molecular markers for SE in tree peony. This study improves our understanding of the molecular mechanisms that underlie SE.

## Figures and Tables

**Table 1:** Summary of Illumina transcriptome sequencing for different periods of somatic embryogenesis.

Sample	Total Number	Total Length (bp)	Mean Length (bp)	N50	GC (%)
S0	61,989	63,967,148	1031	1673	40.83
S1	50,923	50,805,120	997	1588	41.34
S2	83,500	68,001,019	814	1417	40.26
S3	57,926	56,509,588	975	1540	41.23
S4	69,093	61,369,650	888	1473	40.84
All-Unigene	131,496	124,701,759	948	1666	40.04

**Table 2:** The annotations of tree peony unigenes against the public databases.

Values	Total	NR	NT	Swiss-Prot	KEGG	KOG	Pfam	GO	Overall
Unigene number	131,496	57,737	48,779	42,056	45,084	44,846	41,037	43,696	66,261
Percentage %	100%	43.91%	37.10%	31.98%	34.29%	34.10%	31.21%	33.23%	50.39%

**Table 3:** Identification of the candidate genes annotated to somatic embryogenesis.

No.	Unigene ID	Gene Name	Gene Name	Sequence Length (bp)	Homology Species & GeneBank Number	CDS Length of Homology Species (bp)
**SAM construction and somatic embryogenesis**						
1	Unigene17677_All	*WUS*	*WUSCHEL*	804	*Durio zibethinus*, XM_022896431.1	1167
2	CL13560.Contig2_All	*KNAT6*	*Homeobox protein knotted-1-like 6*	1388	*Vitis vinifera*, XM_002263277.3	957
3	Unigene15414_All	*LET6*	*Homeobox protein knotted-1-like LET6*	1468	*Vitis riparia*, XM_034844888.1	969
4	Unigene22766_All	*CUC2*	*CUP-SHAPED COTYLEDON 2*	1603	*Vitis vinifera*, XM_010645872.2	1092
5	CL13321.Contig1_All	*CUC2*	*CUP-SHAPED COTYLEDON 2*	1499	*Vitis vinifera*, XM_010645872.2	1167
6	CL12019.Contig1_All	*ATH1*	*Homeobox protein ATH1*	2488	*Vitis riparia*, XM_034850593.1	6672
7	CL1776.Contig3_All	*ATH1*	*Homeobox protein ATH1*	5137	*Vitis vinifera*, XM_010650028.2	1773
8	CL14012.Contig2_All	*ATHB15*	*Homeobox-leucine zipper protein*	3224	*Vitis riparia*, XM_034842502.1	2538
9	CL1733.Contig3_All	*MAIN*	*MAIN-LIKE 1-like*	747	*Papaver somniferum*, XM_026565731.1	1881
10	CL9670.Contig1_All	*LEU*	*LEUNIG-like isoform X2*	799	*Vitis riparia*, XM_034843924.1	2601
11	CL5605.Contig4_All	*REV*	*REVOLUTA*	3615	*Vitis riparia*, XM_034832108.1	2535
12	CL5605.Contig5_All	*REV*	*REVOLUTA*	2302	*Vitis riparia*, XM_034832108.1	2535
13	Unigene28422_All	*WOX9*	*WUSCHEL-related homeobox 9*	1695	*Theobroma cacao*, XM_018122721.1	1131
14	CL2926.Contig4_All	*WRKY2*	*WRKY transcription factor 2 isoform X1*	2666	*Vitis riparia*, XM_034822984.1	2241
15	Unigene25819_All	*WOX11*	*WUSCHEL related homeobox 11*	1058	*Theobroma cacao*, CM001883.1	840
16	Unigene1508_All	*WOX4*	*WUSCHEL-related homeobox 4*	887	*Paeonia suffruticosa*, KJ466969.1	1065
17	Unigene9596_All	*BBM*	*BABY BOOM*	2615	*Theobroma cacao*, XM_018121249.1	2139
18	Unigene5205_All	*DCAF1*	*DDB1- and CUL4-associated factor homolog 1*	5941	*Vitis vinifera*, XM_010650165.2	5904
19	CL1628.Contig3_All	*ATML1*	*Homeobox-leucine zipper protein MERISTEM L1*	2844	*Vitis vinifera*, XM_002266652.3	2181
20	CL13336.Contig1_All	*SERK2*	*Somatic embryogenesis receptor kinase 2*	2395	*Olea europaea* var. sylvestris, XM_022994562.1	1884
21	CL13336.Contig2_All	*SERK1*	*Somatic embryogenesis receptor kinase 1*	3775	*Citrus unshiu*, AB115767.1	1866
22	CL3929.Contig2_All	*SERK4*	*Somatic embryogenesis receptor kinase 4-like*	1969	*Durio zibethinus*, XM_022881312.1	2049
23	CL4277.Contig6_All	*SERK1*	*Somatic embryogenesis receptor kinase 1*	2465	*Citrus unshiu*, AB115767.1	1866
24	CL11145.Contig4_All	*ABI3*	*B3 domain-containing transcription factor ABI3-like*	3013	*Pistacia vera*, XM_031422459.1	2751
25	Unigene27385_All	*ABI3*	*B3 domain-containing transcription factor ABI3-like*	2293	*Pistacia vera*, XM_031422459.1	2751
26	Unigene66623_All	*FUS3*	*B3 domain-containing transcription factor FUS3-like*	1431	*Durio zibethinus*, XM_022896099.1	879
**Asymmetric cell division**						
27	CL13462.Contig5_All	*SCR*	*SCARECROW*	2869	*Carica papaya*, XM_022041537.1	2367
28	Unigene4284_All	*SCR*	*SCARECROW*	872	*Carica papaya*, XM_022041537.1	2367
29	CL13774.Contig1_All	*SHR*	*SHORT-ROOT*	2515	*Vitis riparia*, XM_034836085.1	1485
**Cell division**						
30	CL529.Contig1_All	*CDC2*	*Cell division control protein 2 homolog C*	1193	*Prunus avium*, XM_021973344.1	915
31	CL5245.Contig6_All	*CDC6*	*Cell division control protein 6 homolog B-like isoform X1*	1797	*Tripterygium wilfordii*, XM_038827626.1	1686
32	CL2953.Contig4_All	*CYCA1-1*	*Cyclin-A1-1 like*	1861	*Actinidia chinensis* var. chinensis, NKQK01000011.1	1485
33	Unigene7857_All	*CYCA2-4*	*Cyclin-A2-4*	1997	*Jatropha curcas*, XM_012217895.3	1467
34	CL2318.Contig2_All	*CYCD1-1*	*Cyclin-D1-1-like*	1404	*Juglans regia*, XM_018990267.2	1083
35	Unigene14153_All	*CYCD4-2*	*Cyclin-D4-2-like*	1741	*Vitis riparia*, XM_034818905.1	1068
36	CL11664.Contig1_All	*MYB3R-4*	*Transcription factor MYB3R-4-like isoform X2*	2040	*Camellia sinensis*, XM_028263128.1	1791
37	CL3905.Contig3_All	*SCC3*	*Sister-chromatid cohesion protein 3 isoform X2*	3910	*Vitis vinifera*, XM_019221250.1	3483
38	CL5482.Contig5_All	*CDK2*	*Cyclin-dependent kinase G-2 isoform X1*	3638	*Vitis vinifera*, XM_019221819.1	2265
39	CL12537.Contig2_All	*CYCA3-1*	*Cyclin-A3-1*	1532	*Vitis riparia*, XM_034818851.1	1098
40	CL8129.Contig1_All	*CYCA3-1*	*Cyclin-A3-1*	1470	*Vitis riparia*, XM_034818851.1	1098
41	CL12075.Contig1_All	*CCNB1-2*	*G2/mitotic-specific cyclin S13-7*	1754	*Vitis vinifera*, XM_002283116.3	1362
**Cell expansion**						
42	CL14446.Contig3_All	*GRF1*	*Growth-regulating factor 1*	2396	*Vitis riparia*, XM_034836315.1	1794
43	CL4841.Contig1_All	*GRF1*	*Growth-regulating factor 1-like*	1822	*Vitis riparia*, XM_034839265.1	1092
44	CL11999.Contig3_All	*GRF3*	*Growth-regulating factor 3*	1574	*Nelumbo nucifera*, XM_010245626.2	1155
45	Unigene21950_All	*GRF3*	*Growth-regulating factor 3*	1261	*Nelumbo nucifera*, XM_010245626.2	1155
46	CL1368.Contig5_All	*GRF4*	*Growth-regulating factor 4 isoform X2*	1660	*Vitis riparia*, XM_034855923.1	1749
47	CL13597.Contig2_All	*GRF8*	*Growth-regulating factor 8*	1775	*Vitis riparia*, XM_034847823.1	1578
**Cell fate determination**						
48	Unigene8884_All	*YAB1*	*Axial regulator YABBY 1*	978	*Theobroma cacao*, XM_007052051.2	639
49	Unigene5866_All	*YAB1*	*Axial regulator YABBY 5*	1180	*Vitis vinifera*, XM_002285292.4	558
50	CL1629.Contig7_All	*RBR*	*Retinoblastoma-related protein-like*	1486	*Nelumbo nucifera*, XM_010251033.2	3093
51	Unigene30474_All	*RBR*	*Retinoblastoma-related protein isoform X2*	2766	*Ricinus communis*, XM_002529942.4	3063
52	Unigene18914_All	*WIP2*	*Zinc finger protein WIP2*	1086	*Vitis vinifera*, XM_002277501.3	1038
53	CL9942.Contig2_All	*WIP2*	*Zinc finger protein WIP2-like*	1378	*Herrania umbratica*, XM_021422800.1	1035
54	CL7208.Contig5_All	*NOV*	*NO VEIN*	8164	*Vitis riparia*, XM_034830903.1	8283

## Data Availability

Not applicable.

## Supplementary Materials

The following supporting information can be downloaded at: https://www.mdpi.com/article/10.3390/ijms231810595/s1.
