# Supplementary material for: A Comparative Transcriptome Analysis Reveals the Molecular Mechanisms That Underlie Somatic Embryogenesis in Peaonia ostii ‘Fengdan’
Source: Int J Mol Sci. 2022 Sep 13;23(18):10595. doi: 10.3390/ijms231810595 (PMC9505998; doi:10.3390/ijms231810595)
Supplement: Supplementary file 1 [file ijms-23-10595-s001.zip › Supplmentary tables and figures/Supplementary figure S1.pdf]

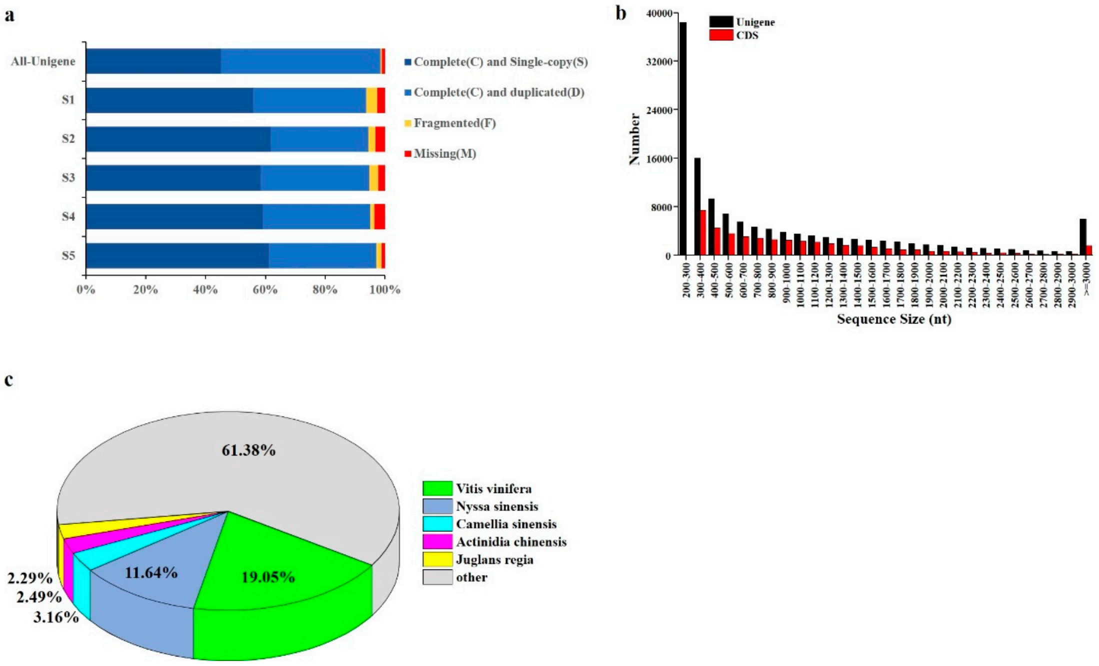

Supplemental S1 Transcriptome assembly and integrity (a), the distribution of the unigene length (b), and the comparison of the unigenes annotated with homologous sequences of other plant species (c).
