# Supplementary material for: A Comparative Transcriptome Analysis Reveals the Molecular Mechanisms That Underlie Somatic Embryogenesis in Peaonia ostii ‘Fengdan’
Source: Int J Mol Sci. 2022 Sep 13;23(18):10595. doi: 10.3390/ijms231810595 (PMC9505998; doi:10.3390/ijms231810595)
Supplement: Supplementary file 1 [file ijms-23-10595-s001.zip › Supplmentary tables and figures/Supplementary figure S2.pdf]

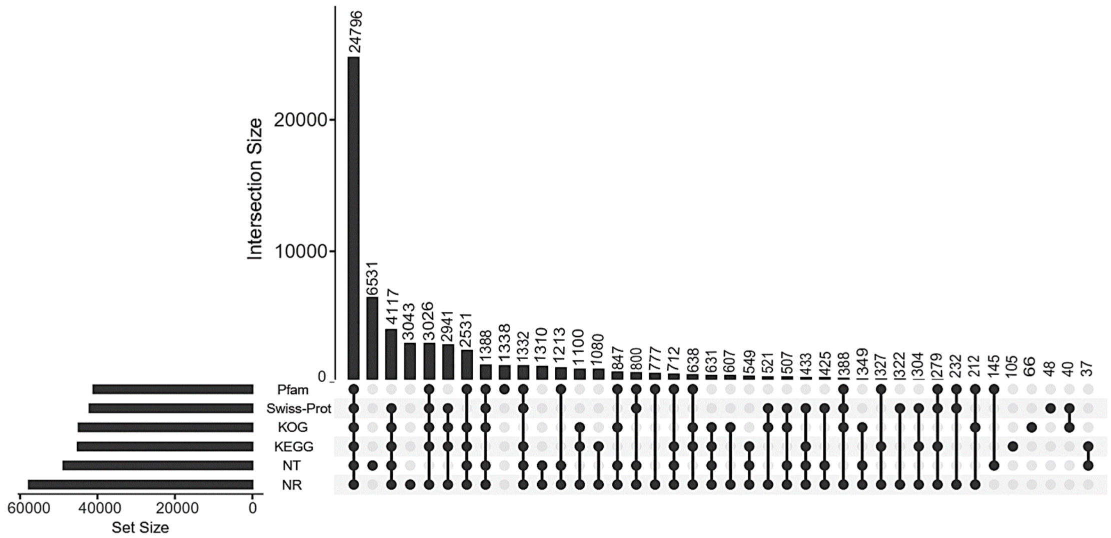

Figure S2. UpSet plot showing the transcripts annotated by the public databases including NCBI non-redundant protein sequences (NR) database, NCBI non-redundant nucleotide sequences (NT) database, Kyoto Encyclopedia of Genes and Genomes(KEGG), Eukaryotic Orthologous Groups of proteins/Clusters of Orthologous Groups of proteins (KOG), Swiss-Prot, and protein structure domain database (Pfam).
