# Supplementary material for: A Comparative Transcriptome Analysis Reveals the Molecular Mechanisms That Underlie Somatic Embryogenesis in Peaonia ostii ‘Fengdan’
Source: Int J Mol Sci. 2022 Sep 13;23(18):10595. doi: 10.3390/ijms231810595 (PMC9505998; doi:10.3390/ijms231810595)
Supplement: Supplementary file 1 [file ijms-23-10595-s001.zip › Supplmentary tables and figures/Supplementary figure S4.pdf]

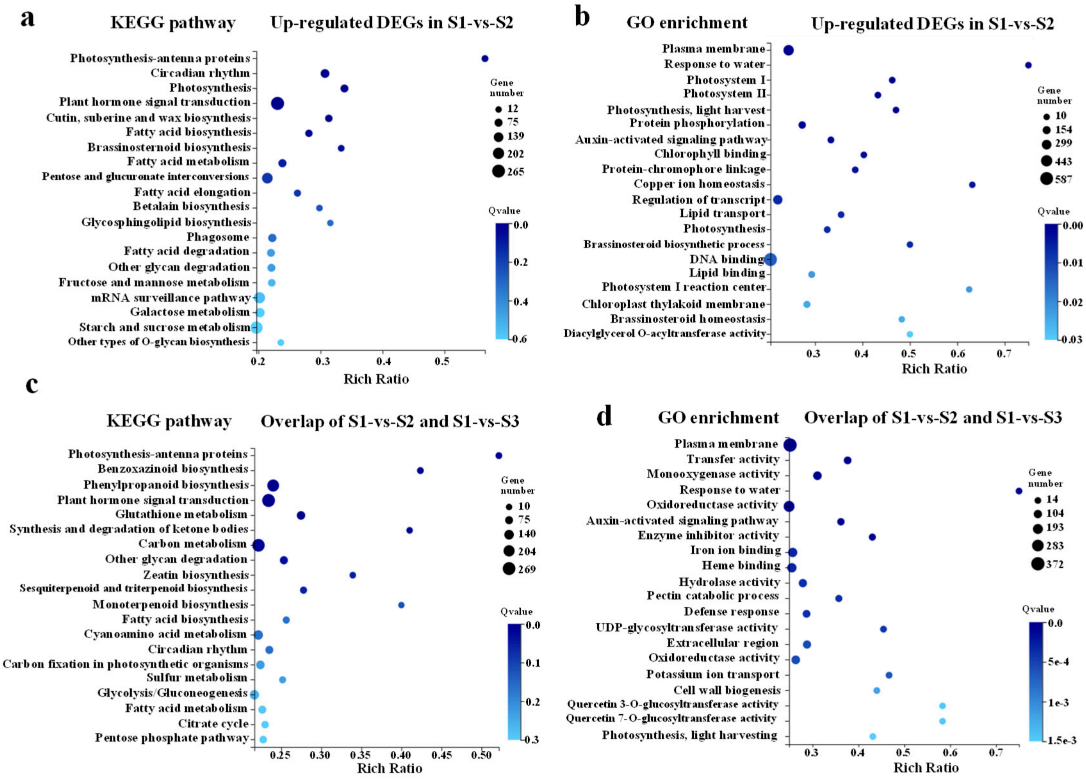

Figure S4. KEGG and GO enrichment analysis of the up-regulated DEGs in S1-vs-S2 and S1-vs-S3 and the overlap DEGs in these two groups. **(a)** KEGG enrichment of the up-regulation DEGs in S1-vs-S2, (**b**) GO enrichment of the up-regulation DEGs in S1-vs-S2, (c) KEGG enrichment of overlap DEGs in S1-vs-S2 and S1-vs-S3, (d) GO enrichment of overlap DEGs in S1-vs-S2 and S1-vs-S3.
