# Supplementary material for: A Comparative Transcriptome Analysis Reveals the Molecular Mechanisms That Underlie Somatic Embryogenesis in Peaonia ostii ‘Fengdan’
Source: Int J Mol Sci. 2022 Sep 13;23(18):10595. doi: 10.3390/ijms231810595 (PMC9505998; doi:10.3390/ijms231810595)
Supplement: Supplementary file 1 [file ijms-23-10595-s001.zip › Supplmentary tables and figures/Supplementary table S3.pdf]

Table S3. Identification of the candidate genes annotated to the putative regulation pathways of somatic embryogenesis.

No.	Unigene ID	Gene name	Gene name	Sequence length(bp)	Homology species & GeneBank number	CDS length of Homology species (bp)
**Auxin pathway**						
1	Unigene16861_All	*GH3.10*	*Indole-3-acetic acid-amido synthetase GH3.10*	1856	*Vitis riparia*, XM_034844950.1	1779
2	Unigene10922_All	*GH3.6*	*Indole-3-acetic acid-amido synthetase GH3.6*	2167	*Populus alba*, XM_035053832.1	1836
3	CL9460.Contig1_All	*YUC10*	*YUCCA10*	1415	*Pistacia vera*, XM_031402923.1	1205
4	CL9657.Contig2_All	*PIN2*	*Auxin efflux carrier*	2788	*Parasponia andersonii*, JXTB01000318.1	1971
5	Unigene10530_All	*PIN1B*	*Auxin efflux carrier component 1b*	2903	*Prunus dulcis*, XM_034369401.1	1794
6	CL2840.Contig1_All	*LAX3*	*Auxin transporter-like protein*	2451	*Jatropha curcas*, XM_012232501.3	1455
7	CL997.Contig3_All	*LAX3*	*Auxin transporter-like protein 2*	1961	*Vitis vinifera*, XM_002277381.3	1470
8	CL9062.Contig2_All	*P23-1*	*Co-chaperone protein p23-1-like*	973	*Juglans regia*, XM_035695460.1	582
9	Unigene8559_All	*BG1*	*BIG GRAIN 1-like B*	1515	*Durio zibethinus*, XM_022900694.1	1158
10	Unigene9560_All	*BG1*	*BIG GRAIN 1-like E*	1344	*Manihot esculenta*, XM_021763941.2	960
11	Unigene21382_All	*GRH1*	*Auxin signaling F-box protein 2*	3571	*Paeonia suffruticosa*, MT702583.1	1719
12	Unigene27194_All	*GRH1*	*Auxin signaling F-box protein 2*	2691	*Paeonia suffruticosa*, MT702583.1	1719
13	Unigene27196_All	*GRH1*	*Auxin signaling F-box protein 2*	2118	*Paeonia suffruticosa*, MT702583.1	1719
14	Unigene40406_All	*GRH1*	*Auxin signaling F-box protein 2*	1351	*Paeonia suffruticosa*, MT702583.1	1719

15	Unigene72333_All	*GRH1*	*Auxin signaling F-box protein 2*	589	*Paeonia suffruticosa*, MT702583.1	1719
16	CL10648.Contig4_All	*TIR1*	*TRANSPORT INHIBITOR RESPONSE 1-like*	3583	*Sesamum indicum*, XM_011084194.2	1746
17	Unigene29603_All	*ARF2*	*Auxin response factor 2*	761	*Malus domestica*， XM_029098909.1	972
18	CL7022.Contig2_All	*ARF2B*	*Auxin response factor 2B*	2687	*Vitis riparia*, XM_034817049.1	2589
19	CL3267.Contig2_All	*ARF4*	*AUXIN RESPONSE 4*	1725	*Juglans microcarpa x Juglans regia*, XM_041141698.1	1461
20	CL11425.Contig2_All	*ARF4*	*Auxin response factor 4 isoform X1*	1623	*Vitis riparia*, XM_034832353.1	2397
21	Unigene7938_All	*ARF4*	*Auxin response factor 4-like*	2745	*Juglans regia*, XM_035695460.1	2247
22	CL8431.Contig9_All	*ARF3*	*Auxin response factor 3*	4157	*Vitis vinifera*, XM_010657162.2	2220
23	CL2228.Contig4_All	*ARF9*	*Auxin response factor 9,*	2860	*Vitis riparia*, XM_034854337.1	2088
24	Unigene377_All	*ARF18*	*Auxin response factor 18-like*	2297	*Nelumbo nucifera*, XM_010255396.2	2184
25	Unigene378_All	*ARF18*	*Auxin response factor 18-like*	1173	*Nelumbo nucifera*, XM_010255396.2	2184
26	Unigene25640_All	*IAA13*	*Auxin-responsive protein IAA13-like*	1067	*Vitis riparia*, XM_034829001.1	966
27	Unigene5418_All	*IAA16*	*Auxin-responsive protein IAA16*	1095	*Theobroma cacao*, XM_007030505.2	750
28	Unigene24531_All	*IAA26*	*Auxin-responsive protein IAA26-like*	2243	*Quercus lobata*, XM_031115396.1	1089
29	CL2908.Contig1_All	*IAA27*	*Auxin-responsive protein IAA27*	1764	*Macadamia integrifolia*, XM_042630785.1	1026
30	CL4761.Contig1_All	*IAA27*	*Auxin-responsive protein IAA27-like*	1471	*Vitis riparia*, XM_034840638.1	1032
31	Unigene8709_All	*IAA31*	*Auxin-responsive protein IAA31*	683	*Fragaria vesca subsp. vesca*, XM_004296983.2	639
32	CL11672.Contig1_All	*IAA9*	*Auxin-responsive protein IAA9-like*	1632	*Vitis riparia*, XM_034835082.1	1143
33	CL15190.Contig5_All	*IAA9*	*Auxin-responsive protein IAA9*	868	*Prunus mume*, XM_008233626.1	1137
34	Unigene74872_All	*IAA19*	*AUX/IAA protein*	519	*Parasponia andersonii*, JXTB01000318.1	618

35	Unigene8208_All	*IAA14*	*Auxin-responsive protein IAA14-like*	1043	*Gossypium hirsutum*, XM_016819048.2	750
36	Unigene27633_All	*AUX6B*	*Auxin-induced protein 6B*	998	*Manihot esculenta*, XM_021745952.2	453
37	Unigene6177_All	*AUX15*	*Auxin-induced protein X15-like*	786	*Populus alba*, XM_035041144.1	246
38	CL14076.Contig2_All	*AUX22B*	*Auxin-induced protein 22B-like*	941	*Juglans microcarpa x Juglans regia*, XM_041137590.1	591
**Cytokinin pathway**						
39	Unigene8674_All	*IPT5*	*Adenylate isopentenyltransferase 5, chloroplastic*	1219	*Coffea eugenioides*, XM_027310009.1	1002
40	Unigene2390_All	*LOG*	*Cytokinin riboside 5′-monophosphate phosphoribohydrolase*	893	*Trema orientale*, JXTC01000550.1	675
41	CL11378.Contig1_All	*LOG3*	*Cytokinin riboside 5′-monophosphate phosphoribohydrolase LOG3*	969	*Momordica charantia*, XM_022289953.1	1002
42	CL13131.Contig1_All	*LOG5*	*Cytokinin riboside 5′-monophosphate phosphoribohydrolase LOG5*	1006	*Prunus mume*, XM_008237887.1	1010
43	Unigene10839_All	*LOG1*	*Cytokinin riboside 5′-monophosphate phosphoribohydrolase LOG1-like isoform X1*	1051	*Pistacia vera*, XM_031414305.1	672
44	Unigene5319_All	*LOG*	*Cytokinin riboside 5′-monophosphate phosphoribohydrolase LOG*	1032	*Corchorus capsularis*, AWWV01012464.1	654
45	CL3091.Contig2_All	*IPT2*	*tRNA dimethylallyltransferase 2*	847	*Quercus suber*, PKMF03010630.1	256
46	CL2238.Contig4_All	*AHK3*	*Histidine kinase 3 isoform X2*	4660	*Carya illinoinensis*, XM_043122764.1	3042
47	Unigene39624_All	*AHK4*	*Histidine kinase 4 isoform X2*	2551	*Vitis riparia*, XM_034828223.1	3141
48	CL15136.Contig2_All	*AHK4*	*Histidine kinase 4 isoform X2*	4070	*Vitis riparia*, XM_034828223.1	3141
49	CL11912.Contig2_All	*AHP2*	*Histidine-containing phosphotransfer*	870	*Citrus clementina*, XM_006436586.2	438

			protein 2 isoform X1			
50	CL1552.Contig1_All	*AHP1*	*Histidine-containing phosphotransfer protein 1*	915	*Vitis vinifera*, XM_002265271.4	453
51	CL3407.Contig3_All	*ORR21*	*Two-component response regulator ORR21*	2530	*Vitis riparia*, XM_034820990.1	1989
52	CL12697.Contig2_All	*ARR1*	*Two-component response regulator ARR1-like isoform X2*	3330	*Vitis riparia*, XM_034830642.1	2043
53	CL5520.Contig1_All	*ARR9*	*Two-component response regulator ARR9*	1179	*Carya illinoinensis*, XM_043085939.1	675
54	CL5885.Contig2_All	*ARR12*	*Two-component response regulator ARR12-like*	2741	*Quercus lobata*, XM_031095425.1	2097
55	Unigene13676_All	*APRR1*	*Two-component response regulator like*	961	*Actinidia chinensis var. chinensis*, NKQK01000008.1	1980
56	CL660.Contig3_All	*APRR1*	*Two-component response regulator-like protein*	2399	*Morus notabilis*, KE345304.1	1791
57	CL660.Contig4_All	*APRR2*	*Two-component response regulator-like APRR2 isoform X*	1912	*Vitis riparia*, XM_034854656.1	1674
58	CL10511.Contig4_All	*APRR7*	*Two-component response regulator-like APRR7*	2140	*Herrania umbratica*, XM_021433410.1	2352
59	CL14817.Contig17_All	*PRR37*	*Two-component response regulator-like PRR37 isoform X2*	3065	*Vitis vinifera*, XM_002281740.3	2358
60	CL6432.Contig1_All	*PUP1*	*Purine permease 1*	1379	*Vitis vinifera*, XM_002285681.4	1050
61	Unigene8674_All	*IPT5*	*Adenylate isopentenyltransferase 5*	1219	*Coffea eugenioides*, XM_027310009.1	1002
62	Unigene2390_All	*LOG*	*Cytokinin riboside 5′-monophosphate phosphoribohydrolase*	893	*Trema orientale*, JXTC01000550.1	675
63	CL11378.Contig1_All	*LOG3*	*Cytokinin riboside 5′-monophosphate*	969	*Momordica charantia*, XM_022289953.1	XM_022289953.1

			phosphoribohydrolase LOG3			
**ABA pathway**						
64	CL86.Contig2_All	*RH3*	*DEAD-box ATP-dependent RNA helicase 3*	3265	*Vitis riparia*, XM_034850244.1	2295
65	Unigene1227_All	*PYL4*	*Abscisic acid receptor PYL4*	1134	*Mangifera indica*, XM_044647129.1	669
66	Unigene24912_All	*PYL4*	*Abscisic acid receptor PYL4*	1744	*Macadamia integrifolia*, XM_042656042.1	660
67	Unigene6464_All	*PYL3*	*Regulatory components of ABA receptor 3*	1488	*Actinidia rufa*, BJWL01000002.1	459
68	Unigene1617_All	*PYL9*	*Abscisic acid receptor PYL9*	942	*Citrus clementina*, XM_006444807.2	561
69	Unigene11665_All	*PYR1*	*Abscisic acid receptor PYR1-like*	999	*Ziziphus jujuba*, XM_016041382.2	654
70	CL3285.Contig3_All	*DPBF3*	*ABSCISIC ACID-INSENSITIVE 5-like protein 2 isoform X2*	1460	*Camellia sinensis*, XM_028264802.1	1002
71	CL11123.Contig2_All	*ABF4*	*ABSCISIC ACID-INSENSITIVE 5-like protein 7*	931	*Juglans microcarpa x Juglans regia*, XM_041154888.1	1272
72	CL4510.Contig1_All	*ABF4*	*ABSCISIC ACID-INSENSITIVE 5-like protein 7*	2132	*Juglans microcarpa x Juglans regia*, XM_041154888.1	1272
73	CL12028.Contig5_All	*ARP1*	*RNA-binding protein ARP1*	1795	*Morus notabilis*, XM_024173535.1	753
**GA pathway**						
74	CL1913.Contig1_All	*GID1c*	*GID1c*	1980	*Paeonia suffruticosa*, MH546122.1	1035
75	CL1374.Contig1_All	*GID1c*	*GID1c*	1897	*Paeonia suffruticosa*, MH546122.1	1035
**Ethylene pathway**						
76	CL15551.Contig3_All	*ACO11*	*1-aminocyclopropane-1-carboxylate*	1435	*Quercus suber*, XM_024032620.1	1113

			oxidase homolog 11-like			
77	CL4421.Contig3_All	*ACO11*	*1-aminocyclopropane-1-carboxylate oxidase homolog 11-like*	1372	*Camellia sinensis*, XM_028254194.1	1926
78	Unigene15908_All	*ACO11*	*1-aminocyclopropane-1-carboxylate oxidase homolog 11*	820	*Jatropha curcas*, XM_037635790.1	1005
79	CL3712.Contig4_All	*ACO1*	*1-aminocyclopropane-1-carboxylate oxidase homolog 1-like*	1305	*Vitis riparia*, XM_034829518.1	1098
80	CL240.Contig1_All	*ACO4*	*1-aminocyclopropane-1-carboxylate oxidase homolog 4-like*	1441	*Quercus suber*, XM_024055251.1	1095
81	CL6909.Contig2_All	*ACO*	*1-aminocyclopropane-1-carboxylate oxidase*	2276	*Paeonia suffruticosa*, FJ855434.1	939
82	CL6123.Contig5_All	*PIF4*	*Transcription factor PIF4*	2221	*Rosa chinensis*, XM_024328547.2	1617
83	Unigene11940_All	*ERF*	*Ethylene-responsive transcription factor ERF098-like*	479	*Juglans regia*, XM_018971599.2	423
84	CL9359.Contig3_All	*ERF*	*Ethylene-responsive transcription factor-like protein*	4859	*Momordica charantia*, XM_022303847.1	591
85	CL2223.Contig3_All	*EIN3*	*Ethylene insensitive 3-like 2 protein*	2406	*Paeonia suffruticosa*, JQ771470.1	1824
**Epigenetic reprogramming**						
86	CL10174.Contig2_All	*SYD*	*Chromatin structure-remodeling complex protein SYD*	7851	*Vitis riparia*, XM_034830593.1	11743
87	Unigene1152_All	*HAT1*	*Histone acetyltransferase type B catalytic subunit*	1760	*Hevea brasiliensis*, XM_021785372.1	1715
88	CL5455.Contig2_All	*HAT3.1*	*Homeobox protein HAT3.1*	3845	*Pistacia vera*, XM_031401101.1	3193
89	CL11525.Contig2_All	*TOP2*	*DNA topoisomerase 2*	4706	*Vitis riparia*, XM_034849047.1	4686

90	CL3272.Contig2_All	*TOP6B*	*DNA topoisomerase 6 subunit B*	1168	*Vitis vinifera*, XM_010652984.2	2281
91	Unigene5813_All	*NRP2*	*NAP1-related protein 2*	1259	*Carya illinoinensis*, XM_043113752.1	1335
92	CL8386.Contig2_All	*HIRA*	*HIRA*	2147	*Vitis riparia*, XM_034831924.1	3193
93	Unigene27426_All	*MCM2*	*DNA replication licensing factor MCM2*	3101	*Quercus lobata*, XM_031104472.1	3271
94	CL2977.Contig5_All	*PKL*	*PICKLE*	2184	*Vitis riparia*, XM_034826486.1	4872
95	CL12991.Contig1_All	*BRM*	*ATP-dependent helicase BRM*	7252	*Vitis vinifera*, XM_002276209.4	7460
96	CL9589.Contig4_All	*HDA9*	*Histone deacetylase 9*	1242	*Vitis riparia*, XM_034832580.1	1618
97	Unigene26959_All	*ASHR1*	*Histone-lysine N-methyltransferase*	1464	*Paeonia lactiflora*, KY535276.1	1440
98	Unigene26961_All	*ASHR1*	*Histone-lysine N-methyltransferase*	1531	*Paeonia lactiflora*, KY535276.1	1440
99	CL11554.Contig2_All	*ATX2*	*Histone-lysine N-methyltransferase*	1370	*Vitis riparia*, XR_004651279.1	3845
100	Unigene75853_All	*ATXR6*	*Histone-lysine N-methyltransferase*	1234	*Camellia sinensis*, XM_028196795.1	1267
101	CL4760.Contig1_All	*ATXR7*	*Histone-lysine N-methyltransferase*	1793	*Quercus suber*, XM_024044167.1	5553
102	CL14179.Contig1_All	*CLF*	*Histone-lysine N-methyltransferase*	2923	*Camellia sinensis*, XM_028200618.1	3229
103	CL3208.Contig6_All	*SUVH4*	*Histone-lysine N-methyltransferase*	2641	*Vitis riparia*, XM_034850652.1	2558
104	Unigene16769_All	*SUVH4*	*Histone-lysine N-methyltransferase*	2214	*Herrania umbratica*, XM_021442212.1	1726
105	Unigene5039_All	*SUVH4*	*Histone-lysine N-methyltransferase*	2419	*Telopea speciosissima*, XM_043847610.1	2403
106	CL1008.Contig3_All	*JMJ703*	*Lysine-specific demethylase*	4658	*Quercus lobata*, XM_031103633.1	4538
107	Unigene16899_All	*DDM1*	*ATP-dependent DNA helicase*	2645	*Vitis riparia*, XM_034827524.1	2764
108	CL9658.Contig5_All	*HAM1*	*Histone acetyltransferase of the MYST family 1*	1397	*Theobroma cacao*，CM001887.1	663

109	CL3517.Contig5_All	*HAC-like 1*	*Histone acetyltransferase*	6325	*Pistacia vera*, XM_031423438.1	3201
110	Unigene10525_All	*HAC1*	*Histone acetyltransferase*	2194	*Vitis vinifera*, XM_010656911.2	5253
111	Unigene19127_All	*HAC-like 1*	*Histone acetyltransferase*	1610	*Camellia sinensis*, XM_028253463.1	4347
112	Unigene10554_All	*JMJ14*	*Lysine-specific demethylase JUMONJI14*	3759	*Vitis vinifera*, QGNW01000059.1	3300
**Central histones in nucleosome**						
113	CL3618.Contig1_All	*H1*	*Histone H1*	1435	*Pisum sativum*, JQ924508.1	600
114	Unigene23750_All	*H2A*	*Histone H2a*	704	*Thalictrum thalictroides*, JABWDY010019253.1	405
115	Unigene72679_All	*H2A*	*Histone H2A*	568	*Trema orientale*, PON33284.1	130
116	Unigene9818_All	*H2A*	*Histone H2A*	651	*Striga asiatica*, BKCP01011070.1	453
117	Unigene50848_All	*H2A.1*	*Histone H2A.1*	484	*Morella rubra*, RXIC02000096.1	426
118	Unigene23810_All	*H2A.1*	*Histone H2A.1-like*	683	*Juglans regia*, XM_018999420.2	438
119	Unigene23012_All	*H2A.1*	*Histone H2A.1-like*	739	*Camellia sinensis*, XM_028259483.1	447
120	Unigene8896_All	*H2A.1*	*Histone H2A.1-like*	734	*Camellia sinensis*	447
121	Unigene20308_All	*H2A.1*	*Histone H2A.1-like*	856	*Camellia sinensis*, XM_028259483.1	447
122	CL14298.Contig1_All	*H2A.3*	*Histone H2A variant 3*	764	*Manihot esculenta*, XM_021737492.2	405
123	Unigene72680_All	*H2A.5*	*Histone H2A.5*	859	*Punica granatum*, XM_031522151.1	420
124	Unigene14888_All	*H2A.5*	*Histone H2A.5*	880	*Vitis riparia*, XM_034851649.1	414
125	CL12587.Contig6_All	*H2AX*	*Histone H2AX*	880	*Vitis vinifera*, XM_002265842.4	420
126	Unigene76811_All	*H2B*	*Histone H2B*	328	*Capsicum annuum*, PHT65196.1	201

127	Unigene71986_All	*H2B.1*	*Histone H2B.1*	676	*Salvia splendens*, XM_042138710.1	447
128	Unigene71987_All	*H2B.1*	*Histone H2B.1*	810	*Salvia splendens*, XM_042138710.1	447
129	Unigene63631_All	*H2B.1*	*Histone H2B.1*	697	*Salvia splendens*, XM_042138710.1	447
130	CL514.Contig10_All	*H2B.7*	*Histone H2B.7*	942	*Benincasa hispida*, XM_039038953.1	429
131	Unigene29887_All	*H2B.7*	*Histone H2B.7*	753	*Momordica charantia*, XM_022297191.1	429
132	CL6675.Contig3_All	*H3.2*	*Histone H3.2*	1428	*Lactuca sativa*, XM_023886701.2	510
133	Unigene18826_All	*H3*	*Histone H3*	579	*Arabidopsis thaliana x Arabidopsis arenosa*, JAEFBK010000003.1	477
134	Unigene8861_All	*CENH3*	*Histone H3-like centromeric protein CENH3*	618	*Cynara cardunculus var. scolymus*, XM_025128344.1	870
135	CL3349.Contig3_All	*CENH3*	*Histone H3-like centromeric protein CENH3*	881	*Helianthus annuus*,	591
136	Unigene7033_All	*H4*	*Histone H4*	878	*Hordeum vulgare*, SDOW01000318.1	489
137	Unigene54617_All	*H4*	*Histone H4-like*	374	*Fragaria vesca subsp. vesca*, XM_004294530.2	453
138	Unigene58283_All	*H4*	*Histone H4-like*	554	*Odocoileus virginianus texanus*, XP_020770838.1	150
Stress related genes						
139	CL1193.Contig2_All	*GALUR*	*D-galacturonate reductase*	1397	*Manihot esculenta*, XM_021743085.2	1143
140	Unigene5397_All	*LGALDH*	*L-galactose dehydrogenase*	1353	*Cannabis sativa*, XM_030631458.1	1509
141	CL10437.Contig1_All	*MDAR5*	*Monodehydroascorbate reductase*	1724	*Quercus suber*, XM_024029577.1	1672
142	CL4597.Contig1_All	*MDAR5*	*Monodehydroascorbate reductase*	1835	*Quercus suber*, XM_024029250.1	1792
143	CL2502.Contig3_All	*PNC1*	*Cationic peroxidase 1*	2037	*Pistacia vera*, XM_031408773.1	
144	Unigene28418_All	*PNC1*	*Cationic peroxidase 1*	1318	*Pistacia vera*, XM_031408773.1	994

145	CL12764.Contig1_All	*AAO*	*L-ascorbate oxidase*	2137	*Carya illinoinensis*, XM_043118778.1	2183
146	Unigene27570_All	*AAO*	*L-ascorbate oxidase*	2218	*Quercus suber*, XM_024050804.1	2166
147	CL15166.Contig2_All	*AAO*	*L-ascorbate oxidase*	2371	*Prunus avium*, XM_021978338.1	1981
148	Unigene10841_All	*AAO*	*L-ascorbate oxidase*	2175	*Pistacia vera*, XM_031427588.1	1942
149	Unigene25370_All	*SMO1-1*	*Methylsterol monooxygenase 1-1*	1510	*Vitis riparia*, XM_034818772.1	1357
150	Unigene24833_All	*PER11*	*Peroxidase 11*	1197	*Prunus avium*, XM_021959465.1	1364
151	CL10036.Contig1_All	*PER12*	*Peroxidase 12*	1255	*Camellia sinensis*, MN594783.1	1080
152	Unigene25803_All	*PER13*	*Peroxidase 12*	1150	*Populus euphratica*, XM_011044586.1	1397
153	Unigene19698_All	*PER17*	*Peroxidase 17*	1349	*Durio zibethinus*, XM_022883589.1	1373
154	CL15223.Contig2_All	*PER2*	*Peroxidase 2*	3427	*Vitis vinifera*, AM468458.2	15226
155	CL1309.Contig2_All	*PER20*	*Peroxidase 20*	1125	*Populus euphratica*, XM_011020320.1	1245
156	Unigene14786_All	*PER21*	*Peroxidase 21*	1255	*Pistacia vera*, XM_031397712.1	1130
157	Unigene73031_All	*PER3*	*Peroxidase 3*	974	*Populus euphratica*, XM_011038705.1	1369
158	CL9105.Contig1_All	*PER4*	*Peroxidase 4*	1325	*Durio zibethinus*, XM_022890270.1	1259
159	Unigene886_All	*PER4*	*Peroxidase 4*	1129	*Ziziphus jujuba*, XM_025070415.1	1207
160	Unigene25358_All	*PER42*	*Peroxidase 42*	1444	*Vitis riparia*, XM_034842604.1	1395
161	CL12453.Contig2_All	*PER43*	*Peroxidase 43*	1064	*Vitis riparia*, XM_034845392.1	1262
162	Unigene77066_All	*PER45*	*Peroxidase 45*	965	*Juglans microcarpa x Juglans regia*, XM_041163513.1	1261
163	Unigene22864_All	*PER47*	*Peroxidase 47*	1282	*Camellia sinensis*, XM_028238787.1	1130
164	CL11055.Contig1_All	*PER64*	*Peroxidase 64*	1159	*Vitis riparia*, XM_034819835.1	1293

165	Unigene28208_All	*PER73*	*Peroxidase 73*	1215	*Quercus lobata*, XM_031067740.1	1054
166	Unigene7518_All	*PERP7*	*Peroxidase P7*	1194	*Medicago truncatula*, XM_003596672.2	957
167	CL15597.Contig2_All	*CYB561A*	*Transmembrane ascorbate ferrireductase 1*	1063	*Populus trichocarpa*, EF145596.1	927
168	CL2142.Contig2_All	*GPX2*	*Glutathione peroxidase 2*	1015	*Malus domestica*, XM_029100877.1	938
169	CL14121.Contig2_All	*GPX5*	*Glutathione peroxidase 5*	757	*Camellia sinensis*, XM_028211627.1	5661
170	Unigene25004_All	*GAT1*	*Thioredoxin M3*	880	*Coffea arabica*, XM_027256712.1	1427
