# Supplementary material for: A Comparative Transcriptome Analysis Reveals the Molecular Mechanisms That Underlie Somatic Embryogenesis in Peaonia ostii ‘Fengdan’
Source: Int J Mol Sci. 2022 Sep 13;23(18):10595. doi: 10.3390/ijms231810595 (PMC9505998; doi:10.3390/ijms231810595)
Supplement: Supplementary file 1 [file ijms-23-10595-s001.zip › Supplmentary tables and figures/Supplementary table S4.pdf]

Table S4. List of primer information for expression analysis of nine somatic embryogenesis related genes

Primer name	The sequence (5'-3')	Length of the product (bp)
qRTWUS-1F	GGTGGTGGGAATAGAAGCC	139
qRTWUS-1R	TGGGAAAGAGTGGAAGGGT	
qRTCUC-1F	CAGCCCTCGTTTTCACCTCT	185
qRTCUC-1R	CGCAAGTTAGCATCTGGGAG	
qRFUS3-1F	TTTCGTCTCCCCATCTGCTC	124
qRFUS3-1R	CCACGGTCATAATAAACCCTTCT	
qRTBBM-1F	GCAAATGTTATAGGGATC	161
qRTBBM-1R	CTTTGTATCATCATTACGC	
qRTSERK-1F	GTTGTAGGTGACTTTGGGTTGG	122
qRTSERK-1R	TCCGATGACTTCCCTGTGG	
qRTWRKY2-1F	GGAGAAGCACCGCTAACC	250
qRTWRKY2-1R	AGCCCTTGCCGAGTATTG	
qRTWOX9-1F	CAAGGGATGAGATAAGGCG	211
qRTWOX9-1R	GATGAGGAAGATGAAGGGC	
qRTWOX4-1F	CCAACAAATAGAACAAATCACT	270
qRTWOX4-1R	ACATCTCACCTCTTCCACACAT	
qRTWOX11-1F	TGAAACGGTGAGGATAAGGAAG	100
qRTWOX11-1R	GTGAGTTGTTGTGCCAGGTGA	
qRTActin-1F	GAGAGATTCCGTTGCCCAG	191
qRTActin-1R	TCCTTGCTCATTCTGTCTGC	
